# Using Mendelian randomization analysis to better understand the relationship between mental health and substance use: a systematic review

**DOI:** 10.1017/S003329172100180X

**Published:** 2021-07

**Authors:** Jorien L. Treur, Marcus R. Munafò, Emma Logtenberg, Reinout W. Wiers, Karin J. H. Verweij

**Affiliations:** 1 Department of Psychiatry, Amsterdam UMC, University of Amsterdam, Amsterdam, the Netherlands; 2 Addiction Development and Psychopathology (ADAPT) Lab, Department of Psychology, University of Amsterdam, Amsterdam, the Netherlands; 3 School of Psychological Science, University of Bristol, Bristol, UK; 4 MRC Integrative Epidemiology Unit, the University of Bristol, Bristol, UK; 5 Center for Urban Mental Health, University of Amsterdam, Amsterdam, the Netherlands

**Keywords:** Systematic review, Mendelian randomization, substance use, smoking, alcohol, cannabis, caffeine, mental disorders, cognitive functioning

## Abstract

**Background:**

Poor mental health has consistently been associated with substance use (smoking, alcohol drinking, cannabis use, and consumption of caffeinated drinks). To properly inform public health policy it is crucial to understand the mechanisms underlying these associations, and most importantly, whether or not they are causal.

**Methods:**

In this pre-registered systematic review, we assessed the evidence for causal relationships between mental health and substance use from Mendelian randomization (MR) studies, following PRISMA. We rated the quality of included studies using a scoring system that incorporates important indices of quality, such as the quality of phenotype measurement, instrument strength, and use of sensitivity methods.

**Results:**

Sixty-three studies were included for qualitative synthesis. The final quality rating was ‘−’ for 16 studies, ‘– +’ for 37 studies, and ‘+’for 10 studies. There was robust evidence that higher educational attainment decreases smoking and that there is a bi-directional, increasing relationship between smoking and (symptoms of) mental disorders. Another robust finding was that higher educational attainment increases alcohol use frequency, but decreases binge-drinking and alcohol use problems, and that mental disorders causally lead to more alcohol drinking without evidence for the reverse.

**Conclusions:**

The current MR literature increases our understanding of the relationship between mental health and substance use. Bi-directional causal relationships are indicated, especially for smoking, providing further incentive to strengthen public health efforts to decrease substance use. Future MR studies should make use of large(r) samples in combination with detailed phenotypes, a wide range of sensitivity methods, and triangulate with other research methods.

## Introduction

Mental disorders have consistently been associated with substance use – in particular cigarette smoking, alcohol drinking, cannabis use, and consumption of caffeinated drinks. Compared to the general population, individuals diagnosed with a mental disorder – or subclinical symptoms – are more likely to smoke (Garey et al., 2020), drink alcohol excessively (Stephen Rich & Martin, 2014), and use cannabis (Satre, Bahorik, Zaman, & Ramo, 2018). For caffeine, there are conflicting findings with high(er) consumption being associated with a lower odds of depression (Grosso, Micek, Castellano, Pajak, & Galvano, 2016) but a higher odds of schizophrenia (Williams & Gandhi, 2008). A key factor in mental disorders is cognitive functioning, the majority of patients suffering from deficits in attention, learning and/or memory (Nieman et al., 2020). In non-clinical populations, poor cognitive functioning has been associated with increased smoking (Campos, Serebrisky, & Castaldelli-Maia, 2016), alcohol drinking (Topiwala & Ebmeier, 2018), and cannabis use (Curran et al., 2016), although for impaired response inhibition specifically there are contradicting findings (Liu et al., 2019b). Although caffeine is often thought to have acute beneficial effects on cognition (Irwin, Khalesi, Desbrow, & McCartney, 2020), there is evidence that contests this (Galindo, Navarro, & Cavas, 2020; Weibel et al., 2020) and its long(er) term effects remain unclear (Cornelis, Weintraub, & Morris, 2020; Panza et al., 2015).

To properly inform public health policy it is crucial to understand the mechanisms underlying associations between poor mental health and substance use. Typically, a distinction is made between three, not mutually exclusive, mechanisms: (1) shared risk factors, (2) causal effects where poor mental health increases substance use, and (3) causal effects where substance use negatively affects mental health. As for mechanism 1, important non-genetic shared risk factors are the death of a loved one (Keyes et al., 2014) or (other) childhood trauma (Setién-Suero et al., 2020). Although note that these seemingly environmental factors might have a heritable component (Sallis et al., 2020). Poor mental health and substance use are substantially heritable and there is evidence for considerable genetic correlation (Abdellaoui, Smit, van den Brink, Denys, & Verweij, 2020; Vink & Schellekens, 2018). However, genetic correlations can also reflect causal relationships. If trait 1 causally affects trait 2, then genetic variants predictive of trait 1 will, indirectly, also predict trait 2 (Kraft, Chen, & Lindström, 2020).

We review evidence from studies that applied ‘Mendelian randomization’ (MR) (Davies, Holmes, & Davey Smith, 2018b; Lawlor, Harbord, Sterne, Timpson, & Davey Smith, 2008) to assess causal effects between poor mental health and substance use. When we talk about a true *causal* effect (e.g. A is causal for B), we imply that if A were to be altered this would lead B to change accordingly. To some extent, MR is analogous to a randomized controlled trial (RCT). Instead of participants being assigned to experimental conditions, MR compares subgroups in the population which are at differing levels of genetic risk for a proposed risk factor. We include MR studies that look at cigarette smoking, alcohol drinking, cannabis use, and/or caffeine consumption in relation to (symptoms of) a mental health disorder or cognitive functioning. Below, we briefly discuss epidemiological and (human) experimental evidence on these relationships and then introduce MR.

### Epidemiological evidence

Causal inference can be attempted by looking at the temporal nature of relationships. For smoking, there is extensive longitudinal evidence that depression (Audrain-McGovern, Leventhal, & Strong, 2015; Mathew, Hogarth, Leventhal, Cook, & Hitsman, 2017) and attention-deficit hyperactivity disorder (ADHD) (van Amsterdam, van der Velde, Schulte, & van den Brink, 2018) are associated with increased odds of smoking initiation and persistence. In the other direction – from smoking to mental health – a systematic review study including 26 studies with a follow-up of between seven weeks and nine years concluded that smoking cessation is followed by reduced depression, anxiety, and stress (Taylor et al., 2014b). Smoking has also been associated with poorer cognitive performance, which improved after cessation (Vermeulen et al., 2018).

For alcohol, a review of 37 longitudinal studies found that (symptoms of) mental disorders in childhood predict an increased odds of alcohol dependence later on in life (Groenman, Janssen, & Oosterlaan, 2017). In the other direction, alcohol dependence and heavy drinking predicted subsequent increases in depressive symptoms, but for heavy drinking, this association did not persist after adjustment for confounders (Li et al., 2020). A systematic review of alcohol interventions reported that alcohol reduction led to a lower prevalence of psychiatric episodes, and improvement of anxiety and depressive symptoms, self-confidence, and mental quality of life (Charlet & Heinz, 2017).

For cannabis use, the few available studies are smaller and the evidence is mixed. A 10-year prospective cohort study in 1395 adolescents found that symptoms of mental disorders (depression, bipolar, and anxiety disorder) increase the odds of cannabis initiation and cannabis use disorder (Wittchen et al., 2007). There was no indication that cannabis causes elevated anxiety symptoms (Twomey, 2017), but substantial evidence to support that it increases the risk of manic symptoms (Gibbs et al., 2015) and psychosis (Gage, Hickman, & Zammit, 2016a). Another study found evidence that cannabis can be beneficial for post-traumatic stress disorder but is associated with short-term cognitive deficits (Walsh et al., 2017).

For caffeine, research has focussed predominately on cognitive functioning or sleep. The largest available systematic review, including 28 studies, concluded that there is some evidence that caffeine is protective against cognitive decline (Panza et al., 2015). Despite the fact that caffeine has stimulating properties which are thought to interfere with sleep acutely, a cohort study in 26 305 adolescents with a follow-up of 4 years found no association between average daily caffeine consumption and sleep duration (Patte, Qian, & Leatherdale, 2018).

Combined, the current epidemiological literature points topotential bi-directional effects between mental health and substance use. However, there are important methodological limitations to consider. First, there may be bias from confounders that were not included in the analysis or measured with considerable error (Gage, Munafò, & Davey Smith, 2016b). Second, reverse causality, where the outcome variable or a precursor of the outcome variable has affected the exposure, can induce spurious associations (Gage et al., 2016).

Family-based studies are better suited for causal inference. Most notable are twin methods. Because monozygotic and dizygotic twins share 100% of their family environment and 100% or 50% of their genetic make-up, respectively, causality can be inferred by looking at within-twin pair differences. For instance, differences in ADHD symptoms were associated with differential progression to daily smoking, cigarettes per day and nicotine dependence in female monozygotic twin pairs, indicating that ADHD causally impacts smoking (Elkins et al., 2018). A study that identified monozygotic twin pairs who were discordant for smoking (one smoked, the other did not), found evidence suggesting that smoking can also causally increase ADHD symptoms (Treur et al., 2015). However, twin methods also have important limitations – there may be bias from confounders that led twins to differ on the exposure as well as on the outcome of interest, and reverse causation cannot be ruled out (McGue, Osler, & Christensen, 2010).

### Experimental evidence from human studies

Experimentally induced stress increased the perceived value of cigarettes in smokers with depressive symptoms (Dahne, Murphy, & MacPherson, 2017). Similarly, when tested after overnight sleep deprivation smokers were more inclined to pick cigarettes over money than when they were tested after a normal night's sleep (Hamidovic & de Wit, 2009). In the other direction, a meta-analysis of 35 clinical trials concluded that participants who were randomly assigned to use nicotine patches to quit smoking experienced more sleep problems than participants assigned not to use them (Greenland, Satterfield, & Lanes, 1998). After randomly assigning 31 smokers to continue smoking and 33 smokers to quit, anxiety and depressive symptoms decreased (more) in the latter group during 3 months follow-up (Dawkins, Powell, Pickering, Powell, & West, 2009).

Among 540 participants randomly assigned to receive different types of treatment for depression there were significant treatment effects on depressive symptoms, but no changes in alcohol consumption (Strid, Hallgren, Forsell, Kraepelien, & Öjehagen, 2019). A considerable amount of work has focussed on cognitive behavioral therapy (CBT) to reduce alcohol consumption. A systematic review including eight RCTs concluded that CBT reduced alcohol use and depressive and/or anxiety symptoms, even when CBT targeted alcohol only (Baker, Thornton, Hiles, Hides, & Lubman, 2012). This could mean that decreases in alcohol use led to improvements in mental health, or that, though not targeted to it specifically, CBT affected depressive/anxiety symptoms.

As reflected in the work described here, only a limited number of causal questions can be answered with experimental designs. Moreover, these questions mostly relate to (relatively) short-term effects. Longer-term effects – for instance, potential effects of prolonged smoking on being diagnosed with a mental disorder, or the impact of lifetime alcohol use on the cognitive decline – cannot be investigated. There are also obvious ethical restrictions; it would not be acceptable to randomize people to initiate or increase their use of an addictive substance.

### Mendelian randomization

**Fig. 1.**
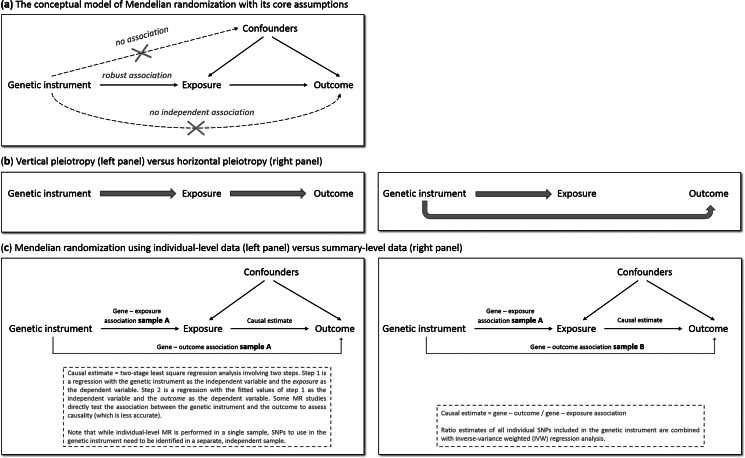
The main principles of Mendelian randomization: (a) the conceptual model indicating the three core assumptions, (b) an illustration of vertical pleiotropy, that which causal inference is based on in a Mendelian randomization analysis, *versus* horizontal pleiotropy, which biases a Mendelian randomization analysis, and (c) an illustration of the framework and methods of Mendelian randomization using individual-level data *versus* summary-level data.

MR has the potential to overcome (some of) the limitations of traditional epidemiological and experimental methods. We will explain MR's rationale by using one specific research question: does smoking (the ‘exposure’ of interest) causally impact depressive symptoms (the ‘outcome’ of interest)? As is the case for practically all human traits (Polderman et al., 2015), individual differences in smoking can partly be explained by genetic differences (Vink, Willemsen, & Boomsma, 2005). Genetic variants robustly associated with smoking have been identified through genome-wide association studies (GWAS) – the most notable variants in nicotinic receptor genes (Liu et al., 2019a). Because the transmission of genetic variants from parents to offspring occurs randomly (Mendel's second law – ‘The law of independent assortment’), there should be minimal bias from confounders and subgroups of differing genetic risk can be thought of as RCT treatment groups. To determine whether smoking causally affects depression, we take genetic variants robustly associated with smoking and test if these also predict higher levels of depressive symptoms. The genetic variants act as proxies for measured smoking behavior, or *instrumental variables* (Davies et al., 2018b). The most commonly used genetic variants are Single Nucleotide polymorphisms (SNPs). MR provides unbiased results if three assumptions are met: (1) the SNPs used as instrumental variables – together referred to as the ‘genetic instrument’ – are robustly associated with the exposure, (2) the genetic instrument is not directly associated with confounders and (3) the genetic instrument is not directly associated with the outcome, apart from any causal effect running through the exposure variable (Fig. 1*a*).

Since the second and third assumptions cannot be known or (exhaustively) tested, sensitivity analyses that assess the robustness of a causal finding are crucial. An important source of bias is pleiotropy, where a genetic variant affects multiple traits. *Vertical* pleiotropy (sometimes called mediated pleiotropy) occurs when a genetic variant affects the exposure and because of that indirectly also affects the outcome. This is not problematic and in fact is what an MR analysis aims to detect. *Horizontal* pleiotropy (sometimes referred to as biological pleiotropy) occurs when a genetic variant affects the outcome independently, not mediated through its effect on the exposure (Fig. 1*b*). This is problematic and could lead to bias.

There are two MR approaches: using individual-level data and using summary-level data from GWAS. Although MR using individual-level data requires a single data set of individuals with genotype data and information on both the exposure and outcome, MR using summary-level data takes summary estimates (i.e. the mean effect size for the genetic variants of interest) from separate GWAS for the exposure and the outcome. The two approaches use different methodology to estimate the causal effect (Burgess, Scott, Timpson, Davey Smith, & Thompson, 2015; Burgess, Small, & Thompson, 2017) (Fig. 1*c*). MR using summary-level data has been the predominant method in recent years and currently has the most (powerful) sensitivity methods.

## Methods

This study was pre-registered at PROSPERO (CRD42019133182; https://www.crd.york.ac.uk/prospero/display_record.php?RecordID=133182). We performed a literature search of Medline, EMBASE, PsycINFO, and Web of Science for published, peer-reviewed papers describing MR of one or more type(s) of substance use in combination with mental health (including diagnoses, subclinical symptoms, and cognitive functioning). We also performed a search of pre-print servers (bioRxiv, medRxiv, and arXiv). We restricted our search to English-language publications (search terms provided in Supplementary Methods). Similar to a recent, high-impact review (Firth et al., 2020), we designed our search to pick up studies that performed analyses referred to as ‘Mendelian randomization’ (or (very) closely related methods such as ‘genetic instrumental variable regression’ (DiPrete, Burik, & Koellinger, 2018)). The final search was performed on 27 February 2020, and a final update of all papers (to incorporate transitions from pre-print to a newer pre-print version or published paper) on 12 April 2021.

We followed PRISMA guidelines in extracting and selecting the data and used a flowchart to document the stages of screening. After a deduplication step, two of the co-authors independently selected potentially eligible studies based on title and abstract, and if necessary in the following step, based on the full text. In case of disagreement between the two main reviewers, this was resolved through discussion with a third co-author.

### Qualitative synthesis

The studies included in this review use a wide range of genetic instruments, phenotypes, and methods. This precluded us from formally combining effect estimates through meta-analysis. Instead, we extracted the most important information from each study, judged the quality based on an extensive set of predetermined criteria, and summarized our findings stratified on the addictive substance.

We developed a scoring system incorporating the factors most important to the validity of an MR study (Supplementary Table S1), based on our collective knowledge of MR and cross-checked with the most recent (still evolving) MR guidelines (Davey Smith et al., 2019). Important indices of quality are phenotype measurement (sample size and quality of the exposure and outcome measurements) and instrument strength (*p* value threshold used to select genetic variants, number of genetic variants included, biological knowledge, *F*-statistic for instrument strength, % variance that the instrument explains). Taking all quality indices into consideration, each study was given a total score of ‘**–**’, ‘**– +**’or‘+’. We considered the total score based on a few key indicators that needed to be satisfied in order for the study to be considered sufficient (**– +** ), most notably: sufficient sample size and sufficient main analytical methods. When, on top of that, a study had used particularly extensive (sensitivity) methods, a total score of (**+**) was given. Two co-authors scored all studies independently and blind from each other, after which they compared their scores. In case of disagreement, a third co-author was consulted and together, all agreed on the final score.

## Results

**Fig. 2.**
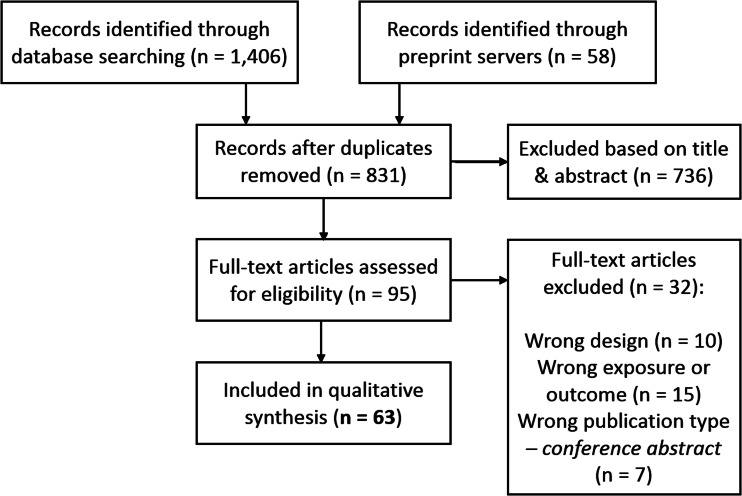
PRISMA flow chart demonstrating the selection of articles to be included for qualitative synthesis.

We identified 1464 potentially relevant records, of which 831 unique (Fig. 2). Of the final 63 studies included in qualitative synthesis, 40 investigated smoking, 24 investigated alcohol, 8 investigated cannabis, and 6 investigated caffeine (some investigated multiple substances; Table 1). The final quality rating was **–** for 16 studies, **– +** for 37 studies, and **+** for 10 studies (Supplementary Tables S2 and S3 for MR using individual-level and summary-level data, respectively). Note that some summary-level studies obtained genetic estimates from partly/largely the same data sets, either for the exposure alone or for both exposure and outcome. This is inherent to MR, as it requires robust, replicated estimates from the largest available GWAS. However, this means that the causal findings presented should not be regarded as (completely) independent. The importance of a particular study and its findings is determined not only on the basis of the data used, but also the quality of the analysis and, importantly, sensitivity methods. If two studies use (almost) exactly the same data sets for exposure and outcome, this is indicated in the text. For a more detailed comparison of data sets see Table 2.

**Table 1.** All Mendelian randomization (MR) studies included for qualitative synthesis, with their identifying information, description of the exposure and outcome variable(s), whether the study used individual-level and/or summary-level data, the total quality rating, and a brief summary of their findings

ID	First author (Year)	Type of substance	Individual- /summary-level data	Exposure variable(s)	Outcome variable(s)	Quality	Finding(s)
1	Zhou et al. (2019a)	Smoking	Summary level	Educational attainment (self-report)	Smoking initiation, cigarettes per day, smoking cessation, age at smoking initiation (all self-report)	–^a^	Evidence for causal, decreasing effects of educational attainment on smoking initiation and cigarettes per day. Evidence for causal, increasing effects of educational attainment on smoking cessation and age at smoking initiation
2	Zeng et al. (2019)	Smoking	Summary level	Educational attainment (self-report)	Smoking initiation (self-report)	– +	Evidence for causal, decreasing effects of educational attainment on smoking initiation. This effect was only modestly attenuated when adjusting for years spent in school
3	Gage et al. (2018)	Smoking	Summary level	Educational attainment (self-report)	Smoking initiation (self-report), cigarettes per day (self-report), smoking cessation (self-report), cotinine levels (measured in the blood)	– +	Evidence for causal, decreasing effects of education on smoking initiation and cigarettes per day. Evidence for causal, increasing effects of education on smoking cessation. Weak evidence for causal, decreasing effects of education on cotinine levels
4	Tillmann et al. (2017)	Smoking	Summary level	Educational attainment (self-report)	Smoking initiation (self-report)	– +	Evidence for causal, decreasing effects of education on smoking initiation
5	Carter et al. (2019)	Smoking	Both	Educational attainment (self-report)	Lifetime smoking (self-report)	– +	Evidence for causal, decreasing effects of education on lifetime smoking
6	Sanderson et al. (2019)	Smoking	Both	Educational attainment (self-report), cognitive functioning (multivariable MR)	Smoking initiation, smoking cessation (all self-report)	+	Evidence for causal, decreasing effects of education on smoking initiation, and evidence for causal, increasing effects of education on smoking cessation. These effects of education are independent of cognitive functioning
7	Gage et al. (2020)	Smoking	Summary level	Smoking initiation (self-report), lifetime smoking (self-report)	Cognitive functioning (fluid intelligence), educational attainment (self-report)	+	Evidence for causal, decreasing effects of smoking initiation and lifetime smoking on educational attainment and cognitive functioning. Results for educational attainment were more robust than results for cognitive functioning
8^b^	Fu et al. (2019)	Smoking	Individual level	Smoking initiation (self-report)	Cognitive functioning (a composite measure of cognitive tests)	–	Evidence for causal, decreasing effects of current smoking on cognitive functioning
9	North et al. (2015)	Smoking	Individual level	Cigarettes per day (self-report)	Cognitive functioning (a general fluid cognitive ability score, derived from a range of different cognitive functioning tests), cognitive decline (% change in continuous cognitive measures from baseline to last available wave)	– +	Overall no consistent evidence for causal effects. Very weak evidence for causal, increasing effects of cigarettes per day on cognitive decline (higher odds of being in the top 25% of cognitive decliners). In never smokers, weak evidence for causal, decreasing effects of smoking on search speed
10^b^	Adams (2019)	Smoking	Summary level	Lifetime smoking (self-report), cognitive functioning (fluid intelligence), neuroticism (self-report)	Lifetime smoking (self-report), cognitive functioning (fluid intelligence), neuroticism (self-report)	– +	Evidence for causal, increasing effects of lifetime smoking on neuroticism. Evidence for causal, decreasing effects of cognitive functioning on lifetime smoking
11	Østergaard et al. (2015)	Smoking	Summary level	Smoking initiation, cigarettes per day (all self-report)	Alzheimer's disease (diagnosis)	– +	Evidence for causal, decreasing effects cigarettes per day on Alzheimer's disease. No clear evidence for causal effects of smoking initiation on Alzheimer's disease
12	Gibson et al. (2019)	Smoking	Both	Smoking initiation, cigarettes per day, smoking cessation, sleep duration, chronotype, insomnia (all self-report)	Smoking initiation, cigarettes per day, smoking cessation; sleep duration, chronotype, insomnia (all self-report)	– +	Evidence for causal, decreasing effects of cigarettes per day on the odds of being a morning person. Weak evidence for causal, increasing effects of insomnia on cigarettes per day, and weak evidence for causal, decreasing effects of insomnia on smoking cessation. No clear evidence for causal effects in any of the other tested relationships
13	Millard et al. (2019)	Smoking	Individual level	Cigarettes per day (self-report)	Chronotype (self-report)	– +	Evidence for causal, decreasing effects of cigarettes per day on the odds of being a morning person
14	Jansen et al. (2019)	Smoking	Summary level	Insomnia (self-report), cigarettes per day (self-report)	Insomnia (self-report), cigarettes per day (self-report)	– +	Evidence for causal, increasing effects of insomnia on cigarettes per day. No clear evidence for causal effects of cigarettes per day on insomnia
15	Lane et al. (2019)	Smoking	Summary level	Insomnia (self-report)	Smoking initiation, age at smoking initiation, cigarettes per day, smoking cessation (all self-report)	– +	No clear evidence for causal effects
16	Bjorngaard et al. (2013)	Smoking	Individual level	Current smoking, cigarettes per day (all self-report)	Anxiety and depression (self-reported Hospital Anxiety and Depression Scale)	– +	Conflicting findings: in the whole sample evidence for causal, increasing effects of smoking on anxiety, but when stratified, effects were very weak in smokers (stronger in former and never smokers). No clear evidence for causal effects of smoking on depression
17	Lewis et al. (2011)	Smoking	Individual level	Smoking status (current, former, never smoker), cigarettes per day (all self-report)	Postnatal depression (self-reported Edinburgh Postnatal Depression Scale)	–	Weak evidence for causal, decreasing effects of smoking status and cigarettes per day on depressed mood during pregnancy
18	Taylor et al. (2014a)	Smoking	Individual level	Cigarettes per day (self-report)	Psychological distress (composite score derived from a range of self-report or symptom scale or diagnosis measures)	– +	No clear evidence for causal effects
19	Skov-Ettrup et al. (2017)	Smoking	Individual level	Cigarettes per day, pack years of cigarettes (all self-report)	Psychological distress (3 questions on stress, fatigue & hopelessness – all self-report)	– +	No clear evidence for causal effects
20	Wootton et al. (2019)	Smoking	Summary level	Lifetime smoking (self-report), smoking initiation (self-report); schizophrenia (diagnosis); major depression (diagnosis)	Lifetime smoking (self-report), smoking initiation (self-report), schizophrenia (diagnosis), major depression (diagnosis)	+	Evidence for causal, increasing effects of smoking initiation and of lifetime smoking on schizophrenia and on depression. Evidence (less strong) for causal, increasing effects of depression on smoking initiation and lifetime smoking, and of schizophrenia on lifetime smoking
21	Vermeulen et al. (2019)	Smoking	Summary level	Lifetime smoking (self-report), smoking initiation (self-report), bipolar disorder (diagnosis)	Lifetime smoking (self-report), smoking initiation (self-report), cigarettes per day (self-report), smoking cessation (self-report); bipolar disorder (diagnosis)	+	Evidence for causal, increasing effects of smoking initiation and of lifetime smoking on bipolar disorder. No clear evidence for causal effects of bipolar disorder on smoking
22^b^	Barkhuizen et al. (2020)	Smoking	Summary level	Smoking initiation (self-report), psychotic experiences (self-report), schizophrenia (diagnosis), major depression (diagnosis), bipolar disorder (diagnosis)	Smoking initiation (self-report); psychotic experiences (self-report), schizophrenia (diagnosis); major depression (diagnosis); bipolar disorder (diagnosis)	– +	Evidence for causal, increasing effects of smoking initiation on major depression, bipolar disorder and cognitive disorganization. Weak evidence for causal, increasing effects of smoking initiation on schizophrenia and on negative symptoms. Weak evidence for causal, increasing effects of schizophrenia on smoking initiation. No clear evidence for causal effects in any of the other tested relationships
23	Wium-Andersen et al. (2015a)	Smoking	Individual level	Cigarettes per day (self-report)	Antipsychotic medication use (national health records), schizophrenia (diagnosis); antidepressant use (national health records), major depression (diagnosis)	– +	Weak evidence for causal, increasing effects of cigarettes per day on antipsychotic medication use and schizophrenia, but no clear evidence for causal effects on depression
24	Byrne et al. (2019)	Smoking	Summary level	Cigarettes per day (self-report)	Schizophrenia (diagnosis)	– +	Evidence for causal, increasing effects of cigarettes per day on schizophrenia
25	Gage et al. (2017b)	Smoking	Summary level	Smoking initiation (self-report), schizophrenia (diagnosis)	Schizophrenia (diagnosis); smoking initiation (self-report), cigarettes per day (self-report), smoking cessation (self-report)	– +	Evidence for causal, increasing effects of smoking initiation on schizophrenia. No clear evidence for causal effects of schizophrenia on smoking
26	Fluharty et al. (2018)	Smoking	Summary level	Childhood aggression (parental report; meta-analysis of continuous study-specific scales) and attention-deficit hyperactivity disorder (diagnosis)	Smoking initiation, age at onset smoking (all self-report)	– +	Evidence for causal, increasing effects of attention-deficit hyperactivity disorder on smoking initiation. No clear evidence for causal effects of attention-deficit hyperactivity disorder on age at onset smoking nor of aggression on smoking
27	Sallis et al. (2019)	Smoking	Both	Extraversion, neuroticism, smoking initiation, cigarettes per day, smoking cessation (all self-report)	Extraversion, neuroticism; smoking initiation, cigarettes per day, smoking cessation (all self-report)	– +	Evidence for causal, increasing effects of neuroticism on cigarettes per day and for causal, increasing effects of extraversion on smoking initiation No clear evidence for causal effects of smoking on extraversion or neuroticism
28^b^	Leppert et al. (2019)	Smoking	Summary level	attention-deficit hyperactivity disorder (diagnosis)	Lifetime smoking (self-report)	–^a^	Evidence for causal, increasing effects of attention-deficit hyperactivity disorder on lifetime smoking
29	Harrison et al. (2020a)	Smoking	Both	Smoking initiation, cigarettes per day, lifetime smoking (all self-report)	Suicidal ideation (self-report)	– +	No clear evidence for causal effects
30	Rosoff et al. (2019)	Alcohol	Summary level	Educational attainment (self-report); frequency of alcohol use (self-report), alcohol drinks per week (self-report), specific alcohol types in drinks per week (self-report), problematic alcohol use (self-reported alcohol use disorders identification test), alcohol use disorder (diagnosis), individual alcohol use disorder symptoms (self-report)	Educational attainment (self-report); frequency of alcohol use (self-report), alcohol drinks per week (self-report), specific alcohol types in drinks per week (self-report), problematic alcohol use (self-reported alcohol use disorders identification test), alcohol use disorder (diagnosis), individual alcohol use disorder symptoms (self-report)	– +	Evidence for causal, decreasing effects of education on total drinks per drinking day, weekly spirits intake, binge drinking, and alcohol use disorder. Evidence for causal, increasing effects of education on alcohol intake frequency, weekly wine intake. In the other direction, evidence for causal, increasing effects of weekly wine and champagne intake and frequency of alcohol use on education, and evidence for causal, decreasing effects of weekly beer and cider intake on education
31	Zhou et al. (2019b)	Alcohol	Summary level	Years of education (self-report)	Alcohol use frequency (self-report), frequency of different types of alcohol use (self-report)	– +	Evidence for causal, increasing effects of educational attainment on alcohol use frequency, frequency of red wine use, and frequency of white wine/champagne use. Evidence for causal, decreasing effects of educational attainment on frequency of beer/cider and spirits
32	Kumari et al. (2014)	Alcohol	Individual level	Alcohol initiation, frequency of alcohol use (all self-report)	Cognitive functioning (word recall, verbal fluency, processing speed tasks)	–	No clear evidence for causal effects
33	Almeida et al. (2014a)	Alcohol	Individual level	Frequency of alcohol use (self-report)	Cognitive impairment (in-person mini-mental state examination)	–	No clear evidence for causal effects
34	Ritchie et al. (2014)	Alcohol	Individual level	Alcohol use in gram per day (self-report)	Cognitive functioning (in-person Moray House Test No. 12)	–	No clear evidence for causal effects of alcohol on cognitive functioning, However, there was an interaction such that individuals with higher genetic ability to process alcohol showed relative improvements in cognitive ability with more consumption, whereas those with low processing capacity showed a negative relationship
35	Au Yeung et al. (2012)	Alcohol	Individual level	Alcohol drinks per day (in-person interview)	Cognitive functioning (10-word list learning task + in-person mini-mental state examination)	–	No clear evidence for causal effects
36	Mahedy et al. (2020)	Alcohol	Summary level	Alcohol drinks per week (self-report)	Working memory, response inhibition, emotion recognition (all in-clinic test assessments)	–	No clear evidence for causal effects
37	Andrews et al. (2020)	Alcohol	Summary level	Alcohol drinks per week (self-report), problematic alcohol use (self-reported Alcohol Use Disorders Identification Test), alcohol use disorder (diagnosis)	Alzheimer's disease (diagnosis), Alzheimer's disease age of onset (diagnosis)	– +	No clear evidence for causal effects of alcohol on Alzheimer's disease diagnosis. Evidence that higher number of alcohol drinks per week causes earlier Alzheimer's disease onset. Contradicting, there was evidence that alcohol use disorder caused later disease onset
38	Nishiyama et al. (2019)	Alcohol	Individual level	Alcohol drinking days per week; cups of coffee per day (all self-report)	Hours of sleep per night (self-report)	–	Evidence that alcohol causes longer sleep duration, no clear evidence for causal effects of coffee on sleep
39	Almeida et al. (2014b)	Alcohol	Individual level	Frequency of alcohol use (self-report)	Depression (self-report on receiving treatment for or being diagnosed with depression; for a subgroup diagnosis obtained from national health records)	–	No clear evidence for causal effects
40	Wium-Andersen et al. (2015b)	Alcohol	Individual level	Alcohol drinks per week (self-report)	Depression (diagnosis obtained from national health records), psychological distress (self-report)	– +	No clear evidence for causal effects
41	Polimanti et al. (2019)	Alcohol	Summary level	Major depression (diagnosis); alcohol use disorder (diagnosis), alcohol use frequency (self-report), alcohol drinks per week (self-report)	Major depression (diagnosis); alcohol use disorder (diagnosis), alcohol use frequency (self-report), alcohol drinks per week (self-report)	+	Evidence for causal, increasing effects of major depression on alcohol use disorder. No clear evidence for causal effects of major depression on the other alcohol use variables, nor for causal effects in the other direction
42	Zhou et al. (2020)	Alcohol	Summary level	Major depression (diagnosis), schizophrenia (diagnosis), bipolar disorder (diagnosis), depressed effect (self-report), neuroticism (self-report), worrying (self-report), insomnia (self-report), cognitive functioning (fluid intelligence), educational attainment (self-report), alcohol use disorder (diagnosis)	Major depression (diagnosis), schizophrenia (diagnosis), bipolar disorder (diagnosis), depressed affect (self-report), neuroticism (self-report), worrying (self-report), insomnia (self-report), cognitive functioning (fluid intelligence), educational attainment (self-report), alcohol use disorder (diagnosis)	+	Evidence for causal, increasing effects of worrying and neuroticism on alcohol use disorder. Evidence for causal, decreasing effects of cognitive functioning and educational attainment on alcohol use disorder. Evidence for causal, decreasing effects of alcohol use disorder and education. No clear evidence for causal effects in any of the other tested relationships
43	Irons et al. (2007)	Alcohol	Individual level	Alcohol initiation, past year use of alcohol, past year drinking index, past year drunkenness index (all self-report), alcohol use disorder (clinical, in-person interview)	Antisocial personality disorder, delinquent behavior inventory (all clinical, in-person interview), exposure to bad peer models (self-report)	–	No clear evidence for causal effects
44	Chao et al. (2017)	Alcohol	Individual level	Alcohol use frequency, alcohol drinks per typical drinking occasion, desire to drink (all self-report)	Externalizing problems (Youth Self-Report), internalizing problems (self-report on Children's Depression Inventory and State-trait Anxiety Inventory)	–	Evidence for causal, increasing effects of alcohol on aggression and attention problems but no clear evidence for effects on delinquency, anxiety, or depression
45	Hodgson et al. (2020)	Cannabis	Summary level	Cannabis initiation (self-report), major depression (diagnosis)	Cannabis initiation (self-report), major depression (diagnosis)	– +	No clear evidence for causal effects
46	Soler Artigas et al. (2019)	Cannabis	Summary level	attention-deficit hyperactivity disorder (diagnosis), cannabis initiation (self-report)	Attention-deficit hyperactivity disorder (diagnosis), cannabis initiation (self-report)	– +	Evidence for causal, increasing effects of attention-deficit hyperactivity disorder on cannabis initiation. No clear evidence causal effects of cannabis initiation on attention-deficit hyperactivity disorder
47	Pasman et al. (2018)	Cannabis	Summary level	Cannabis initiation (self-report), schizophrenia (diagnosis)	Cannabis initiation (self-report), schizophrenia (diagnosis)	– +	Evidence for causal, increasing effects of schizophrenia on cannabis initiation. Weak evidence for causal, increasing effects of cannabis initiation on schizophrenia
48	Vaucher et al. (2018)	Cannabis	Summary level	Cannabis initiation (self-report)	Schizophrenia (diagnosis)	– +	Evidence for causal, increasing effects of cannabis initiation on schizophrenia
49	Gage et al. (2017a)	Cannabis	Summary level	Cannabis initiation (self-report); schizophrenia (diagnosis)	Cannabis initiation (self-report); schizophrenia (diagnosis)	– +	Evidence for causal, increasing effects of schizophrenia on cannabis initiation. Weak evidence for causal, increasing effects of cannabis initiation on schizophrenia
50	Zhou et al. (2018)	Coffee	ndividual level	Cups of coffee per day (self-report)	Cognitive functioning (composite global cognition & memory scores, derived from a range of different cognitive functioning tests)	– +	No clear evidence for causal effects
51	Treur et al. (2018)	Coffee	Summary level	Cups of coffee (self-report), plasma caffeine (measured in blood), caffeine metabolic ratio (measured in blood), sleep duration (self-report), chronotype (self-report), insomnia (self-report)	Cups of coffee (self-report), plasma caffeine (measured in blood), caffeine metabolic ratio (measured in blood); sleep duration (self-report), chronotype (self-report), insomnia (self-report)	– +	Weak evidence for causal, decreasing effects of higher plasma caffeine levels on the odds of being a morning person. No clear evidence for causal effects in any of the other tested relationships
52	Kwok et al. (2016)	Coffee	Summary level	Cups of coffee per day (self-report)	Major depression (diagnosis), Alzheimer's disease (diagnosis)	– +	No clear evidence for causal effects
53	Ding et al. (2019)	Multiple: smoking alcohol	Individual level	Years of education (self-report)	Current smoking (self-report); alcohol drinking days per week (self-report)	–	Evidence for causal, decreasing effects of educational attainment on current smoking. No clear evidence for causal effects of educational attainment on alcohol drinking days per week
54^b^	Yuan et al. (2020a)	Multiple: smoking alcohol :	Summary level	Educational attainment (self-report); cognitive functioning (fluid intelligence)	Age at smoking initiation, cigarettes smoked per day, alcohol drinks per week (all self-report)	–^a^	Evidence for causal, increasing effects of educational attainment on age at onset smoking and decreasing effect on cigarettes per day, the effect remained the same when adjusted for cognitive functioning. Evidence for causal, increasing effects of cognitive functioning on age at onset smoking and decreasing effect on cigarettes per day, but when adjusted for educational attainment, the effect was largely attenuated Evidence for causal, increasing effects of educational attainment on alcohol drinks per week, when adjusted for cognitive functioning, this effect was attenuated. Evidence for causal, increasing effects of cognitive functioning on alcohol drinks per week, the effect remained the same when adjusted for educational attainment
55	Davies et al. (2019)	Multiple: smoking alcohol	Both	Years of school (self-report), cognitive functioning (fluid intelligence)	Smoking initiation, current smoking; alcohol use frequency (all self-report)	+	Evidence for causal, increasing effects of cognitive functioning on alcohol use frequency. Evidence for causal, decreasing effects of years of school on smoking initiation and current smoking
56^b^	Davies et al. (2018a)	Multiple: smoking alcohol	Both	Years of education (self-report)	Alcohol use frequency; smoking initiation, current smoking (all self-report)	+	Evidence for causal, increasing effects of more years of education on alcohol use frequency, and evidence for causal, decreasing effects of more years of education on smoking initiation and current smoking
57	Harrison et al. (2020b)	Multiple: smoking alcohol	Both	Alcohol drinks per week; smoking initiation, lifetime smoking (all self-report)	Education (self-reported university degree status), loneliness (self-report)	– +	Evidence for causal, decreasing effects of lifetime smoking and smoking initiation on education. No clear evidence for causal effects in any of the other tested relationships
58	Mahedy et al. (2021)	Multiple: smoking cannabis	Both	Smoking initiation (self-report); cannabis initiation (self-report)	Working memory, response inhibition, emotion recognition (all in-clinic test assessments)	–	No clear evidence for causal effects
59	Andrews et al. (2021)	Multiple: smoking alcohol	Summary level	Alcohol drinks per week (self-report), problematic alcohol use (self-reported alcohol use disorders identification test); smoking initiation (self-report), cigarettes per day (self-report)	Alzheimer's disease (diagnosis), Alzheimer's disease age at onset (disorder)	+	No clear evidence for causal effects
60	Larsson et al. (2017)	Multiple: smoking alcohol coffee	Summary level	Smoking initiation, cigarettes per day, smoking cessation; alcohol drinks per week; cups of coffee per day (all self-report)	Alzheimer's disease (diagnosis)	– +	Weak evidence for a causal, decreasing effect of cigarettes smoked per day on Alzheimer's disease. Weak evidence for a causal, increasing effect of coffee on Alzheimer's disease. No clear evidence for causal effects of smoking initiation, smoking cessation or alcohol
61	Wootton et al. (2020)	Multiple: smoking alcohol	Summary level	Alcohol drinks per week (self-report), alcohol use disorder (diagnosis), smoking initiation (self-report), loneliness (self-report)	Alcohol drinks per week (self-report), alcohol use disorder (diagnosis), smoking initiation (self-report), cigarettes per day (self-report), smoking cessation (self-report); loneliness (self-report)	– +	Weak evidence for causal, increasing effects of loneliness on smoking initiation and cigarettes per day, weak evidence for causal, decreasing effects of loneliness on smoking cessation. Strong evidence for an effect such that smoking initiation increases loneliness. No clear evidence for causal effects in any of the other tested relationships
62	Lim et al. (2020)	Multiple: alcohol cannabis	Summary level	Cannabis initiation (self-report), alcohol use disorder (diagnosis)	Non-suicidal self-harm (NSSH), suicidal self-harm (SSH) (self-report)	– +	No clear evidence for causal effects
63	Treur et al. (2019)	Multiple: smoking alcohol cannabis coffee	Summary level	Smoking initiation (self-report); alcohol drinks per week (self-report), problematic alcohol use (self-reported Alcohol Use Disorders Identification Test), alcohol use disorder (diagnosis); cannabis initiation (self-report); cups of coffee per day (self-report), attention-deficit hyperactivity disorder (diagnosis)	Smoking initiation (self-report), cigarettes per day (self-report) smoking cessation (self-report), lifetime smoking (self-report), alcohol drinks per week (self-report), problematic alcohol use (self-reported alcohol use disorders identification test), alcohol use disorder (diagnosis), cannabis initiation (self-report), cups of coffee per day (self-report), attention-deficit hyperactivity disorder (diagnosis in adulthood)	+	Evidence for causal, increasing effects of attention-deficit hyperactivity disorder on smoking initiation, cigarettes per day, smoking cessation and cannabis initiation. Weak evidence for causal, increasing effects of attention-deficit hyperactivity disorder on alcohol use disorder. No clear evidence for causal effects of attention-deficit hyperactivity disorder on the other alcohol measures nor on cups of coffee per day In the other direction, weak evidence for causal, increasing effects of smoking initiation on attention-deficit hyperactivity disorder risk

a This score pertains to the relationship that is of interest to the current systematic review, and not necessarily the whole study. For instance, it may be that in the study as a whole (more) extensive MR sensitivity methods were performed but for the causal estimate of interest no sensitivity methods were applied (e.g. when smoking is merely used as a mediator in a multivariable MR study).

b Pre-print publication (not peer-reviewed) obtained from bioRxiv.org, medRxiv.org or arXiv.org.

Note that the quality rating is based on a number of key indices, the most important being: phenotype measurement (sample size, quality of the exposure measurement, quality of the outcome measurement), instrument strength (*p* value threshold used to select genetic variants, number of genetic variants included, biological knowledge, *F* statistic for instrument strength, % variance that the instrument explains), and analytical factors (type of main analysis, whether or not basic sensitivity analyses were applied, whether or not additional sensitivity analyses were applied). Combined, these indices were weighted to come to a complete quality score (see Supplementary Table S1). A few important notes regarding this weighting of the evidence: (1) where absolute thresholds were used to judge the quality of a particular aspect of the study (e.g. sample size), it should be noted that these are somewhat arbitrary and were merely used to provide an indication of quality. (2) With regard to ‘phenotype measurement,’ a very well measured phenotype in a moderate sample size may be just as powerful as a more superficially measured phenotype in a very large sample. However, in case of very small sample sizes (e.g. *n* = 180 such as in the study by Irons et al., 2007) even an extremely thoroughly measured phenotype will not lead to a high total score. (3) With regard to ‘instrument strength,’ when a study uses a single genetic variant that explains a relatively large amount of the variance and for which there is good biological knowledge, the fact that only one SNP was used is not necessarily problematic. For example, this is the case for SNP rs1051730 in the nicotinic acetylcholine receptor *CHRNA5/A3/B4* gene cluster – each additional risk allele increases smoking heaviness with one additional cigarette smoked per day (Katikireddi, Green, Taylor, Davey Smith, and Munafò, 2018).

**Table 2.** All Mendelian randomization (MR) studies included for qualitative synthesis, with their identifying information, description of the data samples used for exposure and outcome variable(s), ancestry of those samples, the independence of the include SNPs, whether or not proxies were used, and whether or not a correction for multiple testing was applied

ID	Author year	GWAS sample exposure variable(s)	Ancestry exposure sample	GWAS sample outcome variable(s)	Ancestry outcome sample	Independence of the SNPs (LD threshold or otherwise)	Proxies used, and if so, LD	Correction multiple testing
1	Zhou et al. (2019a)	Okbay et al. (2016), *N* = 293 723	European	Thorgeirsson et al. (2010) (Tobacco and Genetics (TAG) consortium), smoking initiation effective-*N* = 72 710, smoking cessation effective-*N* = 41 278	European	Independent SNPs as reported in exposure GWAS were selected	Yes, LD *r*^2^ *>* 0.8	None
2	Zeng et al. (2019)	Okbay et al. (2016), *N* = 293 723; Lee et al. (2018), *N* = 1 131 881	European	Thorgeirsson et al. (2010) (TAG consortium) effective-*N* = 72 710	European	Independent SNPs as reported in exposure GWAS were selected	No	None
3	Gage et al. (2018)	Okbay et al. (2016), *N* = 305 072 (Discovery and replication sample, without 23andme)	European	Thorgeirsson et al. (2010) (TAG consortium), smoking initiation effective-*N* = 72 710, cigarettes per day – *N* = 38 181, smoking cessation effective-*N* = 41 278; Ware et al. (2016) – *N* = 4548	European	Independent SNPs as reported in exposure GWAS were selected	Yes, LD *r*^2^ *>* 0.9	None
4	Tillmann et al. (2017)	Okbay et al. (2016), *N* = 349 306	European	Thorgeirsson et al., 2010 (TAG consortium) effective-*N* = 72 710	European	LD *r*^2^ < 0.1	Yes, LD *r*^2^ *>* 0.8	None
5	Carter et al. (2019)	Individual-level MR: UKB (UK Biobank), *N* = 318 147; Summary-level MR: Lee et al. (2018), *N* = 1 131 881	European	Individual-level MR: UKB, *N* = 318 147; Summary-level MR: Wootton et al. (2019), *N* = 462 690	European	LD *r*^2^ < 0.1 education; LD *r*^2^ < 0.001 smoking	No	None
6	Sanderson et al. (2019)	Individual-level MR: UKB, *N* = 120 050; Summary-level MR: Lee et al. (2018), *N* = 1 131 881	European	Individual-level MR: UKB, *N* = 120 050; Summary-level MR: Thorgeirsson et al. (2010) (TAG consortium) smoking initiation effective-*N* = 72 710; smoking cessation effective-*N* = 41 278	European	LD *r*^2^ < 0.001	No	None
7	Gage et al. (2020)	Liu et al. (2019a (GSCAN consortium) without UK Biobank (since it is not explicitly mentioned that these were excluded, it is assumed that 23andme data were *included*), smoking initiation, *N* = 848 460; Wootton et al. (2019), *N* = 462 690	European	Okbay et al. (2016), *N* = 293 723; Cognitive functioning UKB (Neale lab GWAS: http://www.nealelab.is/uk-biobank), *N* = 117 131	European	Independent SNPs as reported in exposure GWAS were selected	No	None
8^a^	Fu et al. (2019)	The Health and Retirement Cohort, *N* = 11 246	European	The Health and Retirement Cohort, *N* = 11 246	European	–	–	None
9	North et al. (2015)	Healthy Aging across the Life Course (HALCyon) consortium, *N* = 22 329	European	HALCyon consortium, *N* = 22 329	European	n.a. (1 SNP)	No	Bonferroni
10^a^	Adams (2019)	Wootton et al., 2019, *N* = 462 690; Cognitive functioning UKB, *N* = 149 051; Okbay et al. (2016), *N* = 170 911	European	Wootton et al. (2019), *N* = 462 690; Cognitive functioning UKB, *N* = 149 051; Okbay et al. (2016), *N* = 170 911	European	LD *r*^2^ < 0.01	No	False Discovery Rate (FDR)
11	Østergaard et al. (2015)	Thorgeirsson et al. (2010) (TAG consortium), smoking initiation effective-*N* = 72 710, cigarettes per day – *N* = 38 181	European	Lambert et al. (2013) (International Genomics of Alzheimer's disease's Project (IGAP)) effective-*N* = 46 668	European	LD *r*^2^ < 0.01	Yes, LD *r*^2^ *>* 0.8	Bonferroni
12	Gibson et al. (2019)	Summary-level MR: Jones et al. (2016) – *N* = 128 266; Hammerschlag et al. (2017), effective-*N* = 92 415; Thorgeirsson et al. (2010) (TAG consortium) smoking initiation effective-*N* = 72 710, cigarettes per day – *N* = 38 181, smoking cessation effective-*N* = 41 278; Individual-level MR: UKB – *N* = 335 708 participants (computed as the biggest sample from Table 1: 184 184 + 118 181 + 33 343)	European	Summary-level MR: Jones et al. (2016), *N* = 128 266; Hammerschlag et al. (2017), effective-*N* = 92 415; Thorgeirsson et al. (2010) (TAG consortium) smoking initiation effective-*N* = 72 710, cigarettes per day – *N* = 38 181, smoking cessation effective-*N* = 41 278; Individual-level MR: UKB – *N* = 335 708 participants (computed as the biggest sample from Table 1: 184 184 + 118 181 + 33 343)	European	LD *r*^2^ < 0.001	Yes, LD *r*^2^ ⩾ 0.8	None
13	Millard et al. (2019)	UKB, *N* = 182 961, never smokers; *N* = 150 831, ever smokers	European	UKB, *N* = 182 961, never smokers; *N* = 150 831, ever smokers	European	n.a. (1 SNP)	No	Bonferonni & FDR (note that this is a PHEWAS)
14	Jansen *et al*. (2019)	Jansen et al. (2019), *N* = 1.3 million; Thorgeirsson et al. (2010) (TAG consortium), *N* = 38 181	European	Jansen et al. (2019), *N* = 1.3 million; Thorgeirsson et al. (2010) (TAG consortium), *N* = 38 181	European	LD *r*^2^ < 0.1	No	Bonferroni
15	Lane et al. (2019)	Frequent insomnia symptoms UKB effective-*N* = 235 787; Any insomnia symptoms UKB effective-*N* = 329 839	European	Thorgeirsson et al. (2010) (TAG consortium) smoking initiation effective-*N* = 72 710, cigarettes per day – *N* = 38 181, smoking cessation effective-*N* = 41 278, age at initiation – *N* = 24 114	European	LD *r*^2^ > 0.8	No	Bonferroni
16	Bjorngaard et al. (2013)	The Trøndelag Health Study (HUNT) Cohort, *N* = 53 601	European	HUNT cohort, *N* = 53 601	European	n.a. (1 SNP)	No	None
17	Lewis et al. (2011)	The Avon Longitudinal Study of Parents and Children (ALSPAC) Cohort, *N* = 6.294	European	ALSPAC cohort, *N* = 6.294	European	n.a. (1 SNP)	No	None
18	Taylor et al. (2014a)	The Causal Analysis Research in Tobacco and Alcohol (CARTA) consortium, *N* = 127 632 (58 176 never smokers, 37 428 former smokers, 32 028 current smokers)	European	CARTA consortium, *N* = 127 632 (58 176 never smokers, 37 428 former smokers, 32 028 current smokers)	European	n.a. (1 SNP)	Yes, rs16 969968 or proxy rs1051730	None
19	Skov-Ettrup et al. (2017)	The Copenhagen General Population study (CGP), *N* = 90 108	European	The Copenhagen General Population study (CGP), *N* = 90 108	European	n.a. (1 SNP)	No	None
20	Wootton et al. (2019)	Wootton et al. (2019) UKB, *N* = 462 690; Liu et al. (2019a) (GSCAN consortium) smoking initiation, *N* = 1.2 million; Schizophrenia working group PGC, 2014 effective-*N* = 111 486; Wray et al. (2018) effective-*N* = 374 559	Predominantly European (small Asian cohorts in schiz GWAS)	Wootton et al. (2019) UKB, *N* = 462 690; Liu et al. (2019a) (GSCAN consortium); smoking initiation, *N* = 599 289; Schizophrenia working group PGC, 2014 effective-*N* = 111 486; Wray et al. (2018) effective-*N* = 141 380	Predominantly European (small Asian cohorts in schiz GWAS)	LD *r*^2^ *<* 0.001	Yes, LD *r*^2^ ⩾ 0.8	None
21	Vermeulen et al. (2019)	Liu et al. (2019a) (GSCAN consortium) smoking initiation, *N* = 1.2 million; Wootton et al. (2019), *N* = 462 690; Stahl et al. (2019) effective-*N* = 49 367	European	Liu et al. (2019a) (GSCAN consortium) smoking initiation, *N* = 1.2 million, cigarettes per day, *N* = 263 954; smoking cessation, *N* = 312 821; Wootton et al. (2019), *N* = 462 690; Stahl et al. (2019) effective-*N* = 49 367	European	Independent SNPs as reported in exposure GWAS were selected	No	None
22^a^	Barkhuizen et al. (2020)	Liu et al. (2019a) (GSCAN consortium) without 23andMe, *N* = 632 802; Pain et al. (2018), *N* = 6297–10 098; Ortega-Alonso et al. (2017), *N* = 3967–4057; UKB (Neale lab: http://www.nealelab.is/uk-biobank), *N* = 157 397; Schizophrenia working group of PGC, 2014 effective-*N* = 111 487; Wray et al. (2018) excluding 23andMe effective-*N* = 156 582; Stahl et al. (2019) effective-*N* = 49 367	Predominantly European (small Asian cohorts in schiz GWAS)	Liu et al. (2019a) (GSCAN consortium) without 23andMe, *N* = 632 802; Pain et al. (2018), *N* = 6297–10 098; Ortega-Alonso et al. (2017), *N* = 3967–4057; UKB (Neale lab: http://www.nealelab.is/uk-biobank), *N* = 157 397; Schizophrenia working group of PGC, 2014 effective-*N* = 111 487; Wray et al. (2018) excluding 23andMe effective-*N* = 156 582; Stahl et al. (2019) effective-*N* = 49 367	Predominantly European (small Asian cohorts in schiz GWAS)	Independent SNPs as reported in exposure GWAS were selected *or* LD *r*^2^ *<* 0.05	No	None for MR (the genetic correlations are corrected for multiple testing)
23	Wium-Andersen et al. (2015a)	Copenhagen General Population Study (CGPS) and Copenhagen City Heart Study (CCHS) cohorts, *N* = 63 296 (23 282 never smokers and 40 014 ever smokers)	European	Copenhagen General Population Study (CGPS) and Copenhagen City Heart Study (CCHS) cohorts, *N* = 63 296 (23 282 never smokers and 40 014 ever smokers)	European	n.a. (1 SNP)	No	None
24	Byrne et al. (2019)	UKB, *N* = 32 510	European	Schizophrenia working group of PGC, 2014 effective-*N* = 99 863 (40 675 cases and 64 643 controls) (note that the PGC schizophrenia working group is referenced but the sample size does not match that in the 2014 PGC publication)	European	Independent SNPs as reported in exposure GWAS were selected	No	None
25	Gage et al. (2017b)	Schizophrenia working group of PGC, 2014 effective-*N* = 111 487; Thorgeirsson et al. (2010) (TAG consortium) effective-*N* = 72 710	Predominantly European (small Asian cohorts in schiz GWAS)	Schizophrenia working group of PGC, 2014 effective-*N* = 111 487; Thorgeirsson et al. (2010) (TAG consortium) smoking initiation effective-*N* = 72 710, cigarettes per day – *N* = 38 181, smoking cessation effective-*N* = 41 278	Predominantly European (small Asian cohorts in schiz GWAS)	LD *r*^2^ *<* 0.9	Yes, LD *r*^2^ *>* 0.9	None
26	Fluharty et al. (2018)	Demontis et al. (2019) effective-*N* = 51 205 (note that the reported sample size implies that a small Asian cohort was included); Pappa et al. (2016) (Early Life Epidemiology consortium (EAGLE)) – *N* = 18 988	Predominantly European (small Asian cohort in attention-deficit hyperactivity disorder GWAS)	Thorgeirsson et al. (2010) (TAG consortium) smoking initiation effective-*N* = 72 710; age at initiation – *N* = 24 114	European	Independent SNPs as reported in exposure GWAS were selected	Yes, LD *r*^2^ ≥ 0.9	None
27	Sallis et al. (2019)	Individual-level MR: UKB, *N* = 273 516; summary-level MR: Thorgeirsson et al. (2010) (TAG consortium) smoking initiation effective-*N* = 72 710, cigarettes per day – *N* = 38 181, smoking cessation effective-*N* = 41 278; Okbay et al. (2016) – *N* = 170 911; Lo et al. (2017) – *N* = 122 886	European	Individual-level MR: UKB – *N* = 273 516; summary-level MR: Thorgeirsson et al. (2010) (TAG consortium) smoking initiation effective-*N* = 72 710, cigarettes per day – *N* = 38 181, smoking cessation effective-*N* = 41 278; Okbay et al. (2016) *– N* = 170 911; Lo et al. (2017) – *N* = 122 886	European	Independent SNPs as reported in exposure GWAS were selected	Yes LD *r*^2^ ≥ 0.8	None
28^a^	Leppert et al. (2019)	Demontis et al. (2019) effective-*N* = 49 017	European	Wootton et al. (2019), *N* = 462 690	European	LD *r*^2^ *<* 0.001	Yes, LD *r*^2^ ≥ 0.9	None
29	Harrison et al. (2020a)	Individual-level MR: UKB, *N* = 463 033/2 (split-sample analyses); Summary-level MR: Liu et al. (2019a) (GSCAN consortium) without UKB and 23andMe – *N* = 249 171; Wootton et al. (2019) – *N* = 463 033	European	Individual-level MR: UKB – *N* = 463 033/2 (split-sample analyses); Summary-level MR: UKB – *N* = effective-*N* = 9661 (2433 cases and 334 766 controls)	European	Independent SNPs as reported in exposure GWAS were selected	Yes, LD *r*^2^ ≥ 0.8	None
30	Rosoff et al. (2019)	Okbay et al. (2016) – *N* = 293 723; Elsworth et al. (2017) alcohol intake frequency – *N* = 462 346, weekly intake – *N* = 326 801; Karlsson Linnér et al. (2019) – *N* = 414 343; Walters et al. (2018) – *N* = 28 657; Sanchez-Roige et al. (2019) – *N* = 121 604	European	Okbay et al. (2016) – *N* = 293 723; Elsworth et al. (2017) alcohol intake frequency – *N* = 462 346, weekly intake – *N* = 326 801; Karlsson Linnér et al. (2019) – *N* = 414 343; Walters et al. (2018) – *N* = 28 657; Sanchez-Roige et al. (2019) – *N* = 121 604	European	LD *r*^2^ = 0.001	It is mentioned a proxy was used for one SNP but not the LD	Bonferroni
31	Zhou et al. (2019b)	Lee et al. (2018) – *N* = 1 131 881	European	UKB, *N* = 334 507	European	Independent SNPs as reported in exposure GWAS were selected	No	None
32	Kumari et al. (2014)	English Longitudinal Study of Aging (ELSA) + Whitehall II study + Health, Alcohol and Psychosocial factors in Easter Europe Study (HAPIEE) combined, *N* = 34 452	European	ELSA + Whitehall II study + HAPIEE combined, *N* = 34 452	European	n.a. (1 SNP)	No	None
33	Almeida et al. (2014a)	The Health in Men Study (HIMS) Cohort, *N* = 3542	Predominantly European	HIMS Cohort, *N* = 3542	Predominantly European	n.a. (1 SNP)	No	None
34	Ritchie et al. (2014)	The Lothian Birth Cohort 1936, *N* = 777	European	The Lothian Birth Cohort 1936, *N* = 777	European	Four SNPs as previously reported in candidate-gene literature	No	None
35	Au Yeung et al. (2012)	The Guangzhou Biobank Cohort Study (GBCS), *N* = 4707	Chinese	GBCS, *N* = 4707	Chinese	n.a. (1 SNP)	No	None
36	Mahedy et al. (2020)	Liu et al. (2019a) (GSCAN consortium), *N* = 941 280	European	ALSPAC Cohort 2500	European	Independent SNPs as reported in exposure GWAS were selected	No	None
37	Andrews et al. (2020)	Liu et al. (2019a) (GSCAN consortium), *N* = 537 349; Sanchez-Roige et al. (2019), *N* = 121 604; Walters et al. (2018) effective-*N* = 34 780	European	Lambert et al. (2013) (IGAP) effective-*N* = 46 668; Huang et al. (2017) effective-*N* = 37 002	European	LD *r*^2^ *<* 0.001	Yes, LD *r*^2^ *>* 0.8	None
38	Nishiyama et al. (2019)	Wakai et al. (2011) (The Japan Multi-Institutional Collaborative Cohort (J-MICC) Study), *N* = 13 618	Japanese	Wakai et al. (2011) (The Japan Multi-Institutional Collaborative Cohort (J-MICC) Study), *N* = 13 618	Japanese	Independent SNPs as reported in exposure GWAS were selected	No	None
39	Almeida et al. (2014b)	HIMS Cohort, *N* = 3873	Predominantly European	HIMS cohort, *N* = 3873	Predominantly European	n.a. (1 SNP)	No	None
40	Wium-Andersen et al. (2015a)	The Copenhagen General Population Study (CGPS) Cohort, *N* = 78 154	European	CGPS Cohort, *N* = 78 154	European	LD *r*^2^ *<* 0.01	No	Bonferroni
41	Polimanti et al. (2019)	Wray et al. (2018) effective-*N* = 389 039; Walters et al. (2018) effective-*N* = 30 053 (note that only unrelated individuals were selected); UKB alcohol use frequency – *N* = 438 308, alcohol use quantity – *N* = 307 098	European	Wray et al. (2018) effective-*N* = 389 039; Walters et al. (2018) effective-*N* = 30 053 (note that only unrelated individuals were selected); UKB alcohol use frequency – *N* = 438 308, alcohol use quantity – *N* = 307 098	European	LD *r*^2^ *<* 0.01	No	Bonferroni
42	Zhou et al. (2020)	Howard et al. (2019) effective-*N* = 684 817; Schizophrenia working group of PGC, 2014 effective-*N* = 111 487; Stahl et al. (2019) effective-*N* = 49 367; Nagel et al. (2018) Neuroticism – *N* = 449 484, depressed affect, – *N* = 357 957, worry – *N* = 348 219; Jansen et al. (2019) – *N* = 1.3 million; Lee et al. (2018) without UKB effective-*N* = 179 185 (based on the first paragraph of the results section MVP phase1, effective-*N* = 114 847 + MVP phase 2, effective-*N* = 37 485 + PGC effective-*N* = 26 853)	Predominantly European (small Asian cohorts in schiz GWAS)	Howard et al. (2019) effective-*N* = 684 817; Schizophrenia working group of PGC, 2014 effective-*N* = 111 487; Stahl et al. (2019) effective-*N* = 49 367; Nagel et al. (2018) Neuroticism – *N* = 449 484, depressed affect – *N* = 357 957, worry – *N* = 348 219; Jansen et al. (2019) – *N* = 1.3 million; Lee et al. (2018) without UKB effective-*N* = 179 185 (based on the first paragraph of the results section MVP phase1, effective-*N* = 114 847 + MVP phase2, effective-*N* = 37 485 + PGC effective-*N* = 26 853)	Predominantly European (small Asian cohorts in schiz GWAS)	Independent SNPs as reported in exposure GWAS were selected	Yes, LD *r*^2^ *>* 0.8	Bonferonni
43	Irons et al. (2007)	McGue et al. (2007) (The Sibling Interaction and Behavior Study), *N* = 180	East Asian (Korean)	McGue et al. (2007) (The Sibling Interaction and Behavior Study), *N* = 180	East Asian (Korean)	n.a. (1 SNP)	No	None
44	Chao et al. (2017)	The BeTwiSt project (adolescents from Beijing), *N* = 1608	Chinese	The BeTwiSt project (adolescents from Beijing), *N* = 1608	Chinese	n.a. (1 SNP)	No	None
45	Hodgson et al. (2020)	Stringer et al. (2016) (International Cannabis Consortium) effective-*N* = 31 933; Wray et al. (2018) effective-*N* = 374 559	European	Stringer et al. (2016) (International Cannabis Consortium) effective-*N* = 31 933; Wray et al. (2018) effective-*N* = 374 559	European	LD *r*^2^ *<* 0.1	No	*p* *<* 0.01 considered significant
46	Soler Artigas et al. (2019)	Demontis et al. (2019 effective-*N* = 49 017; Stringer et al. (2016) (International Cannabis Consortium) effective-*N* = 31 933	European	Demontis et al. (2019 effective-*N* = 49 017; Stringer et al. (2016) (International Cannabis Consortium) effective-*N* = 31 933	European	LD *r*^2^ *<* 0.05	No	None
47	Pasman et al. (2018)	Schizophrenia working group of PGC, 2014 effective-*N* = 111 486; International Cannabis Consortium effective-*N* = 180 934	Predominantly European (small Asian cohorts in schiz GWAS)	Schizophrenia working group of PGC, 2014 effective-*N* = 111 486; International Cannabis Consortium effective-*N* = 180 934	Predominantly European (small Asian cohorts in schiz GWAS)	LD *r*^2^ *<* 0.001	Yes, LD *r*^2^ ≥ 0.8	None
48	Vaucher et al. (2018)	Stringer et al., 2016 (International Cannabis Consortium) effective-*N* = 31 933	European	Schizophrenia working group of PGC, 2014 effective-*N* = 78 227 (note that this sample size is lower than that of the original GWAS, and it was not stated how this subsample was selected)	European (unclear whether the Asian cohorts were included)	10 leading SNPs (not genome-wide significant) from the exposure GWAS, no criteria for independence stated	No	None
49	Gage et al. (2017a)	Stringer et al. (2016) (International Cannabis Consortium) effective-*N* = 31 933; Schizophrenia working group of PGC 2014 effective-*N* = 111 486	Predominantly European (small Asian cohorts in schiz GWAS)	Stringer et al. (2016) (International Cannabis Consortium) effective-*N* = 31 933; Schizophrenia working group of PGC 2014 effective-*N* = 111 486	Predominantly European (small Asian cohorts in schiz GWAS)	*r*^2^ *<* 0.9 (LD was corrected for with correlation matrix)	Yes, LD *r*^2^ *>* 0.9	None
50	Zhou et al. (2018)	Meta-analysis of 10 European cohorts (the 1958 British birth cohort (1958BC), UKB, Mothers of Avon Longitudinal Study of Parents and Children (ALSPAC-M), Northern Finland Birth Cohorts 1966 (NFBC1966), Cardiovascular Risk in Young Finns Study (YFS), Helsinki Birth Cohort Study (HBCS), Prospective Investigation of the Vasculature in Uppsala Seniors (PIVUS), Uppsala Longitudinal Study of Adult Men (ULSAM), Swedish twin registry (STR), and TwinGene), *N* = 415 530 (of which 300 760 coffee consumers)	European	Meta-analysis of 10 European cohorts (1958BC, ALSPAC-M, NFBC1966, YFS, HBCS, PIVUS, ULSAM, STR, and TwinGene), *N* = 415 530 (of which 300 760 coffee consumers)	European	Independent SNPs as reported in exposure GWAS were selected	Yes, LD *r*^2^ 1.0 and 0.97 for two SNPs	None
51	Treur et al. (2018)	Cornelis et al. (2015) (Caffeine Genetics Consortium) – *N* = 91 462; Cornelis et al. (2016) – *N* = 9876; Jones et al. (2016) – *N* = 128 266; Hammerschlag et al. (2017) effective-*N* = 92 415	European	Cornelis et al. (2015) (Caffeine Genetics Consortium), *N* = 91 462; Cornelis et al. (2016), *N* = 9876; Jones et al. (2016), *N* = 128 266; Hammerschlag et al. (2017) effective-*N* = 92 415	European	LD *r*^2^ *<* 0.001	Yes, LD *r* ≥ 0.8	None
52	Kwok et al. (2016)	Cornelis et al. (2015) (Caffeine Genetics Consortium) – *N* = 129 788 (note that the reported sample size implies that the trans-ethnic data was used)	Predominantly European (~6% African American)	Major Depressive Disorder Working Group of PGC, 2013 effective-*N* = 18 755; Lambert et al. (2013) effective-*N* = 46 668	European	Independent SNPs as reported in exposure GWAS were selected	Yes, LD *r*^2^ *>* 0.8	Bonferroni
53	Ding et al. (2019)	Health and Retirement Study (HRS) – *N* = 3935	Not clearly indicated (representative of *>*50 year-olds in the US)	Health and Retirement Study (HRS) *N* = 3935	Not clearly indicated (representative of *>*50 year-olds in the US)	Independent SNPs as reported in exposure GWAS were selected, after which some were excluded to prevent horizontal pleiotropy	No	None
54^a^	Yuan et al. (2020a)	Lee et al. (2018), *N* = 1 131 881; Savage et al. (2018), *N* = 269 867	European	Liu et al. (2019a) (GSCAN consortium) alcohol drinks per week, *N* = 941 280; age at onset smoking, *N* = 341 427; cigarettes per day, *N* = 337 334	European	Independent SNPs as reported in exposure GWAS were selected	No	None
55	Davies et al. (2019)	Individual-level MR: UKB, *N* = 93 135; Summary-level MR: Okbay et al. (2016), *N* = 293 723; Hill et al. (2019), *N* = 248 723	European	Individual-level MR: UKB, *N* = 93 135 Summary-level MR: UKB smoking initiation effective-*N* = 136 760, current smoking effective-*N* = 46 573	European	Independent SNPs as reported in exposure GWAS were selected, further clumped with LD *r*^2^ *<* 0.01	No	None
56^a^	Davies et al. (2018a)	Individual-level MR: UKB – *N* = 315 436; Summary-level MR: Okbay et al. (2016) – *N* = 293 723	European	Individual-level MR: UKB – *N* = 315 436; Summary-level MR: not clear from the manuscript, note that the summary-level analyses were used as a sensitivity analysis to check for pleiotropy and were not the main aim	European	LD *r*^2^ *<* 0.001	Yes, LD *r*^2^ = 1	None
57	Harrison et al. (2020b)	Individual-level MR: UKB *N* = 336 997 [*N* = 336 997/2 (split sample) for lifetime smoking]; Summary-level MR: Liu et al. (2019a) (GSCAN consortium) without UKB and 23andme smoking initiation – *N* = 249 171, alcohol drinks per week – *N* = 226 223; UKB Lifetime smoking – *N* = 336 997 (*N* = 336 997/2 (split sample))	European	Individual-level MR: UKB – *N* = 336 997; Summary-level MR: UKB Lifetime smoking – *N* = 336 997 [*N* = 336 997/2 (split sample)]	European	Independent SNPs as reported in exposure GWAS were selected	Yes, LD *r*^2^ *>* 0.8	Bonferonni
58	Mahedy et al. (2021)	Liu et al. (2019a) (GSCAN consortium) effective-*N* = 1 220 901; Pasman et al. (2018) (International Cannabis Consortium) – *N* = 184 765	European	ALSPAC Cohort, *N* = 3232	European	Independent SNPs as reported in exposure GWAS were selected	No	None
59	Andrews et al. (2021)	Sanchez-Roige et al. (2019) – *N* = 141 932; Liu et al. (2019a) (GSCAN consortium) smoking initiation effective-*N* = 1 220 901, alcohol drinks per week – *N* = 941 280, cigarettes per day – *N* = 337 334	European	Lambert et al. (2013) effective-*N* = 46 670; Kunkle et al. (2019) effective-*N* = 57 692; Huang et al. (2017) – *N* = 40 255	European	LD *r*^2^ *<* 0.001	Yes, LD *r*^2^ ⩾ 0.8	FDR
60	Larsson et al. (2017)	Thorgeirsson et al. (2010) (TAG consortium) smoking initiation effective-*N* = 72 710, cigarettes per day *N* = 38 181, smoking cessation effective-*N* = 41 278;. Jorgenson et al. (2017), *N* = 71 071; Cornelis et al. (2015), *N* = 91 462	European	Lambert et al. (2013) effective-*N* = 46 668	European	LD *r*^2^ *<* 0.2	Yes, LD *r*^2^ *>* 0.9	Bonferroni (0.05/24 test = 0.002), *<*0.05 was reported as ‘suggestive evidence’)
61	Wootton et al. (2020)	Abdellaoui et al. (2019) – *N* = 511 280; Liu et al. (2019a, 2019b) (GSCAN consortium) without UKB and without 23 and me smoking initiation effective-*N* = 244 920, alcohol drinks per week – *N* = 226 223; Walters et al. (2018) effective-*N* = 34 780	European	Abdellaoui et al. (2019) – *N* = 511 280; Liu et al. (2019a, 2019b) (GSCAN consortium) without UKB and without 23andme smoking initiation effective-*N* = 244 920, cigarettes per day – *N* = 249 171, smoking cessation effective-*N* = 142 612, alcohol drinks per week – *N* = 226 223; Walters et al. (2018) effective-*N* = 34 780	European	Independent SNPs as reported in exposure GWAS were selected; for instruments at threshold *p* *<* 1 × 10^−5^, LD *r*^2^ *<* 0.01	Yes, LD *r*^2^ ≥ 0.8	None
62	Lim et al. (2020)	Stringer et al. (2016) (International Cannabis Consortium) effective-*N* = 31 933; Walters et al. (2018), effective-*N* = 34 780	European	UKB – *N* = 125 742	European	LD *r*^2^ *<* 0.001	No	None
63	Treur et al. (2019)	Liu et al. (2019a) (GSCAN consortium) smoking initiation effective-*N* = 1 220 901, alcohol drinks per week – *N* = 941 280; Sanchez-Roige et al. (2019) – *N* = 121 604; Walters et al. (2018) effective-*N* = 34 779; Pasman et al. (2018) (International Cannabis Consortium) effective-*N* = 180 934; Demontis et al. (2019) effective-*N* = 49 017; Cornelis et al. (2016) – *N* = 91 462	European	Liu et al. (2019a) (GSCAN consortium) excluding 23andme smoking initiation effective-*N* = 632 783, cigarettes per day – *N* = 263 954, smoking cessation – *N* = 312 821, alcohol drinks per week – *N* = 537 341; Sanchez-Roige et al. (2019) – *N* = 121 604; Walters et al. (2018) effective-*N* = 34 779; Pasman et al. (2018) (International Cannabis Consortium) effective-*N* = 180 934; Demontis et al. (2019) – *N* = 15 548 (only adults included); Cornelis et al. (2016) – *N* = 91 462	European	Independent SNPs as reported in exposure GWAS were selected	No	None (note the authors explain how they define strength of evidence)

a Pre-print publication (not peer-reviewed) obtained from bioRxiv.org, medRxiv.org or arXiv.org.

Note that the complete references to the samples listed under ‘GWAS sample exposure variable(s)’ and ‘GWAS sample outcome variable(s)’ can be found in the original publications (1–63).

### Cigarette smoking

#### Cognitive traits

There was consistent evidence that higher educational attainment decreases the odds of initiating smoking (Carter et al., 2019; Davies et al., 2018a; Davies et al., 2019; Ding, Barban, & Mills, 2019; Gage, Bowden, Davey Smith, & Munafo, 2018; Sanderson, Davey Smith, Bowden, & Munafò, 2019; Tillmann et al., 2017; Zeng et al., 2019; Zhou et al., 2019a), increases the age at smoking initiation (Yuan, Xiong, Michaëlsson, Michaëlsson, & Larsson, 2020a; Zhou et al., 2019a), increases smoking heaviness, and decreases the odds of quitting (Gage et al., 2018; Sanderson et al., 2019; Zeng et al., 2019; Zhou et al., 2019a). One study triangulated self-report measures with cotinine (a metabolite of nicotine) in blood samples and found weak evidence that higher educational attainment causes lower cotinine levels (Gage et al., 2018). There was considerable overlap among the data sets used (Table 2). Two studies based their education-to-smoking estimate on the same data sets, one testing whether smoking mediated the effects of education on coronary heart disease, and the other whether smoking mediated the effects of education on lung cancer (Tillmann et al., 2017; Zhou et al., 2019a). There was strong evidence that higher general cognitive ability decreases lifetime smoking (Adams, 2019), but no clear evidence for effects on smoking initiation or cessation (Davies et al., 2019). Two multivariable MR studies found that causal effects of education on smoking were not mediated by cognitive ability (Davies et al., 2019; Sanderson et al., 2019).

Substantially fewer studies looked at causal effects ofsmoking on cognitive functioning. There was consistent evidence that smoking initiation and lifetime smoking decrease educational attainment (Gage et al., 2020; Harrison et al., 2020b), and weaker evidence that they decrease cognitive ability (Gage et al., 2020). Two other studies found no clear evidence for causal effects of smoking initiation on cognitive functioning (Adams, 2019; North et al., 2015), but note that the analysis by Gage et al. (2020) was superior (+ *v.* – +). There was also no clear evidence that smoking affects working memory, response inhibition, or emotion recognition, but these analyses were likely underpowered (Mahedy et al., 2021). A single analysis (rated –) reported that current smoking increases the odds of cognitive impairment (Fu, Faul, Jin, Ware, & Bakulski, 2019). Two studies found weak evidence that smoking decreases the odds of Alzheimer's disease (Larsson et al., 2017; Østergaard et al., 2015), but a more recent analysis, rated as superior (+ *v.* – +), found no effects of smoking on Alzheimer's disease (Andrews et al., 2021). The seemingly protective effect of smoking is likely survival bias – smokers who do not die from smoking-related diseases are less prone to diseases making them less likely to develop Alzheimer's disease. Of note, smoking *initiation* is not an ideal measure – there is accumulating evidence that genetic variants associated with this phenotype are horizontally pleiotropic (Khouja, Wootton, Taylor, Smith, & Munafò, 2020). A conclusion on the causal effects of smoking can more reliably be made by testing the effects of smoking heaviness.

#### Sleep problems

There was weak evidence that insomnia increases smoking heaviness and decreases cessation from two studies (Gibson, Munafò, Taylor, & Treur, 2019; Jansen et al., 2019), but not from a third (Lane et al., 2019). In contrast, there was no clear evidence that sleep duration impacts smoking (Gibson et al., 2019). There was particularly strong evidence that smoking heaviness impacts chronotype, decreasing the odds of being a morning person (Gibson et al., 2019; Millard, Munafò, Tilling, Wootton, & Davey Smith, 2019), but no clear evidence that smoking influences insomnia risk or sleep duration (Gibson et al., 2019; Jansen et al., 2019).

#### Internalizing/mood disorders

There was some evidence for causal, increasing effects of depression (Wootton et al., 2019), feelings of loneliness (Wootton et al., 2020), and neuroticism (Sallis, Smith, & Munafo, 2019) on smoking behavior. Adams (2019), using a larger data set for the outcome than Sallis et al. (2019), did not find clear evidence that neuroticism affects smoking. There was no clear evidence for causal effects of depression or bipolar disorder on smoking (Barkhuizen, Dudbridge, & Ronald, 2020; Vermeulen et al., 2019). Most studies also tested effects in the other direction. Earlier studies showed no clear evidence for causal effects (Bjorngaard et al., 2013; Skov-Ettrup, Nordestgaard, Petersen, & Tolstrup, 2017; Taylor et al., 2014a; Wium-Andersen, Orsted, & Nordestgaard, 2015a), with one exception: a small study (*n* = 6294) of low-rated quality reporting that smoking decreased depression during pregnancy (Lewis et al., 2011). More recent studies, employing much larger samples, found strong evidence for causal, increasing effects of smoking initiation and lifetime smoking on depression and bipolar disorder risk (Barkhuizen et al., 2020; Vermeulen et al., 2019; Wootton et al., 2019). There was weak evidence that smoking initiation increases feelings of loneliness (a phenotype closely related to depression) from one study (Wootton et al., 2020), but no such evidence from another (Harrison et al., 2020b). One study reported weak evidence that smoking initiation decreases neuroticism (Sallis et al., 2019), whereas another, better-powered study found that lifetime smoking increases neuroticism (Adams, 2019). Finally, there was no clear evidence that smoking causally impacts suicidal ideation (Harrison et al., 2020a).

#### Externalizing disorders

There was strong evidence that ADHD liability increases smoking initiation, smoking heaviness and lifetime smoking, and decreases cessation (Fluharty, Sallis, & Munafò, 2018; Leppert et al., 2019; Sallis et al., 2019; Treur et al., 2019). There was no clear evidence that aggression causally affects smoking, but this analysis was likely underpowered (Fluharty et al., 2018). One study also tested reverse effects, reporting weak evidence that smoking initiation increases ADHD risk, but with important cautionary notes about the pleiotropic nature of the initiation measure (Treur et al., 2019).

#### Psychotic disorders

Multiple studies reported evidence (ranging from weak to strong) that smoking causally increases schizophrenia risk (Barkhuizen et al., 2020; Byrne et al., 2019; Gage et al., 2017b; Wium-Andersen et al., 2015a, ; Wootton et al., 2019). In the other direction there was no clear evidence for causal effects of liability to schizophrenia on smoking from one study (Gage et al., 2017a, 2017b), and some evidence for such effects from two recent, better-powered studies with largely overlapping samples (Barkhuizen et al., 2020; Wootton et al., 2019).

### Alcohol use

#### Cognitive traits

There was strong evidence that higher educational attainment increases alcohol use frequency (Davies et al., 2018a; Davies et al., 2019; Rosoff et al., 2019; Zhou et al., 2019a, 2019b) and wine intake (Rosoff et al., 2019; Zhou et al., 2019a, 2019b), whereas it decreases beer/cider intake (Zhou et al., 2019a, 2019b), and the risk of binge-drinking and alcohol use disorder (Rosoff et al., 2019; Zhou et al., 2020). Ding et al. (2019) did not find clear evidence for causality from education to alcohol use, but this analysis was likely underpowered. There was also evidence that general cognitive ability increases alcohol use frequency (Davies et al., 2019) and decreases the risk of alcohol use disorder (Zhou et al., 2020). In the other direction, Rosoff et al. (2019) found weak evidence that higher alcohol use decreases educational attainment, whereas another study did not (Harrison et al., 2020b). A third, high-quality rated study found that liability to alcohol use disorder negatively impacts educational attainment (Zhou et al., 2020). Note that GWAS of current alcohol use have largely been performed in adults, reflecting alcohol use after maximum educational attainment occurred for most. Although the genetic instrument may also reflect alcohol use at younger ages, this needs to be taken into account. There was no clear evidence that drinking more alcohol impacts cognition, but this was based on (very) small, low-quality rated studies (Almeida, Hankey, Yeap, Golledge, & Flicker, 2014a, 2014b; Au Yeung et al., 2012; Kumari et al., 2014; Mahedy et al., 2020; Ritchie et al., 2014). There were contradicting findings for Alzheimer's disease, with one study finding no causal effects of alcohol (Larsson et al., 2017) and another finding that while a higher number of drinks caused an earlier onset of Alzheimer's disease, alcohol use disorder caused a later onset (Andrews, Goate, & Anstey, 2020). The latter likely reflects survival bias.

#### Sleep problems

There was some evidence that drinking more alcohol per week increases sleep duration, but this was based on only one, low-quality rated study (Nishiyama et al., 2019).

#### Internalizing/mood disorders

A recent, particularly large study reported strong evidence that major depressive disorder (MDD) liability increases alcohol use disorder risk (Polimanti et al., 2019). Similarly, there was evidence that worrying and neuroticism increase alcohol use disorder risk (Zhou et al., 2020). There was no clear evidence that feelings of loneliness affect alcohol use (disorder) (Wootton et al., 2020). In the other direction, there was no clear evidence that alcohol use (disorder) causally impacts internalizing symptoms (Almeida et al., 2014a, 2014b; Chao, Li, & McGue, 2017; Lim et al., 2020; Polimanti et al., 2019; Wium-Andersen, Orsted, Tolstrup, & Nordestgaard, 2015b; Wootton et al., 2020; Zhou et al., 2020).

#### Externalizing disorders

There was weak evidence that ADHD liability increases alcohol use disorder risk (Treur et al., 2019). In the other direction, there was some evidence that higher alcohol use frequency increases aggression and attention problems from one, small (*n* = 1608) low-rated analysis (Chao et al., 2017), and no evidence for causal effects on antisocial behavior from another very small (*n* = 180) low-rated analysis (Irons, McGue, Iacono, & Oetting, 2007).

#### Psychotic disorders

There was no clear evidence for causal effects, in either direction, between alcohol use disorder and schizophrenia risk (Zhou et al., 2020).

### Cannabis use

#### Cognitive traits

There was no evidence for causal effects from cannabis initiation to cognitive functioning (Mahedy et al., 2021).

#### Internalizing disorders

There was neither clear evidence for causal effects in either direction between cannabis initiation and MDD (Hodgson et al., 2020), nor was there evidence for causal effects from cannabis initiation to self-harm (Lim et al., 2020).

#### Externalizing disorders

There was evidence that ADHD liability increases cannabis initiation without clear evidence for the reverse (Soler Artigas et al., 2019; Treur et al., 2019).

#### Psychotic disorders

Out of eight studies that included cannabis, three looked at schizophrenia. One tested causality from cannabis initiation to schizophrenia risk only, finding evidence for an increasing effect (Vaucher et al., 2018). Two other studies tested causal effects in both directions and found weak evidence that cannabis initiation increases schizophrenia risk and strong evidence that schizophrenia liability increases the odds of cannabis initiation (Gage et al., 2017a, 2017b; Pasman et al., 2018).

### Caffeine consumption

#### Cognitive traits

There was weak evidence that higher coffee consumption increases Alzheimer's risk from one study (Larsson et al., 2017), but no clear evidence from another (Kwok, Leung, & Schooling, 2016). There was also no clear evidence for causal effects of coffee on general cognitive functioning (Zhou et al., 2018).

#### Sleep problems

There was weak evidence that higher plasma caffeine levels decrease the odds of being a morning person, but no clear evidence for causal effects between self-reported caffeine consumption and sleep duration, insomnia, or chronotype (Treur et al., 2018).

#### Internalizing disorders

There was no clear evidence for causal effects between caffeine consumption and ADHD, in either direction (Treur et al., 2019),

#### Externalizing disorders

There was no evidence for causal effects of caffeine consumption on depression (Kwok et al., 2016).

## Discussion

We conducted the first systematic review of MR studies investigating causal relationships between mental health and substance use. From a total of 63 studies, we can draw important conclusions regarding if and how mental health and substance use are causally related.

Smoking was the most investigated, resulting in particularly strong evidence that higher educational attainment *causally* decreases smoking (lower risk of initiating, smoking fewer cigarettes, and more likely to quit). Although smoking prevalence has rapidly decreased in the past two decades, this decline has been most prominent among those with high educational attainment, leading to an increasing (health) gap (Agaku, Odani, Okuyemi, & Armour, 2020). The causal role of education we report is important for policy-makers going forward. Interestingly, causal effects from education are neither mediated by cognitive ability (Sanderson et al., 2019) nor were there clear evidence that cognitive ability by itself affects smoking (Adams, 2019; Davies et al., 2019). The studies included in this review cannot determine exactly why educational attainment affects smoking. Smoking initiation usually occurs during adolescence, at which time the home environment and peer influences are important. Adolescents in lower educational groups tend to experience lower levels of parental involvement, parental monitoring, and self-perceived social competence, factors associated with a higher odds of initiating smoking (Mahabee-Gittens, Xiao, Gordon, & Khoury, 2013; Simons-Morton, 2002). As for smoking heaviness and difficulty quitting, causal mechanisms may involve job opportunities that depend on educational attainment. A lower education often leads to jobs characterized by low skill discretion, high psychological demands and high physical exertion, potentially leading to stress and smoking to cope (Dobson, Gilbert-Ouimet, Mustard, & Smith, 2018a).

Another striking pattern was that of bi-directional, increasing effects between smoking and mental disorders. There was more robust evidence that smoking causally increases the odds of mental disorders than vice versa – most notably for depression, bipolar disorder, and schizophrenia. This concurs with accumulating evidence from longitudinal cohort studies (Taylor et al., 2014b) and animal research (Jobson et al., 2019) indicating neuropsychiatric effects of smoking. A causal mechanism may be that nicotine binds to nicotinic acetylcholine receptors in the brain, given that these are involved in regulating central nervous system pathways relevant to mental disorders (Berk et al., 2011). There is some evidence that repeated nicotine exposure can lead to desensitization of these receptors (Mineur & Picciotto, 2009). Inflammation and oxidative stress induced by toxic compounds from inhaled cigarette smoke is another potential mechanism (Berk et al., 2011). Our conclusion that smoking is detrimental to the brain warrants increased efforts to prevent (heavy) substance use. For individuals with a mental disorder, it implies that smoking cessation may be beneficial to alleviate symptoms. This is an important message given that smokers in this population are not always encouraged to quit (Taylor et al., 2020a). Although not an easy task, it should be communicated to health professionals that there are effective ways to help smokers with a co-morbid mental disorder quit.

A higher education increased alcohol use frequency but decreased the risk of problematic use. Those with higher education tend to drink alcohol more often but spread across multiple drinking occasions, and without developing a dependency. Those with lower education, on the other hand, are at increased odds of developing a problematic relationship with alcohol. This pattern of opposite effects was recently also highlighted in a study that computed genetic correlations and reported high alcohol use frequency to be genetically correlated with higher socio-economic status and lower risk of psychiatric disorders, whereas high alcohol consumption quantity was genetically correlated with lower socio-economic status and higher psychiatric disorder risk (Marees et al., 2019). Similar to smoking, it could be that excessive alcohol use is a way to cope with job stress (Dobson, Ibrahim, Gilbert-Ouimet, Mustard, & Smith, 2018b). There was also consistent evidence that mental disorders increase (problematic) alcohol use, without strong effects in the other direction. The latter implies that observational findings indicating that alcohol use increases mental disorders were due to confounding and/or reverse causality. Indeed, associations between heavy drinking and subsequent increases in depressive symptoms disappeared after adjustment for confounders (Li et al., 2020). It should be noted that this is in contrast to clinical observations where in the *short-term*, treating alcohol use disorder makes pre-existing depression symptoms disappear (Charlet & Heinz, 2017). This discrepancy may be because MR assesses ‘lifetime’ (*longer-term*) effects of alcohol on mental health (Labrecque & Swanson, 2019), and the fact that only a small proportion of those with an alcohol use disorder will receive treatment [*<*9% (Mark, Kassed, Vandivort-Warren, Levit, & Kranzler, 2009)]. In sum, the current MR literature suggests that co-morbidity between poor mental health and alcohol use is primarily the result of alcohol being used as a type of ‘self-medication.’

There was stronger evidence that liability to schizophrenia increases the odds to initiate cannabis, than that cannabis initiation increases schizophrenia risk, as also indicated recently by others (Gillespie & Kendler, 2021). However, these results should be regarded as tentative, given that the genetic instrument for schizophrenia was more powerful than that for cannabis use, and more insightful analyses, with measures of cannabis use frequency, have not yet been performed. This is an important direction for future MR studies, now that such large-scale cannabis studies are becoming available (Hines, Treur, Jones, Sallis, & Munafò, 2020).

For caffeine, the predominantly studied relationships were with cognitive functioning and sleep. Overall, there was no clear evidence that a high average intake of caffeine (negatively or positively) affects cognitive measures or sleep. This is consistent with recent evidence that average (high) caffeine intake does not necessarily result in changes in alertness or sleep patterns, due to the fact that adaptation occurs after repeated intake (Weibel et al., 2020).

### Limitations

Although our scoring system was carefully designed [using the collective experience of the authors and the tentative, developing STROBE-MR ("Strengthening the Reporting of Observational Studies in Epidemiology using Mendelian Randomization") guidelines (Davey Smith et al., 2019)] it should be noted that it was not previously validated. As for the included MR studies, while the more recent were sufficiently powered and some included thorough sensitivity methods and triangulation, many earlier studies were low-quality. In the coming years, it will be important to extend and strengthen the current evidence through MR studies that combine better-powered data sets, preferably with more fine-grained phenotypes and extensive sensitivity methods. An important focal point for smoking and cannabis use as exposure variables is to not only investigate initiation, but also the heaviness of use. There is ample evidence that measurements of initiation can introduce bias due to horizontal pleiotropy and reverse causality (Khouja et al., 2020; Li et al., 2020; Treur et al., 2019; Yuan, Yao, & Larsson, 2020b). Another important addition to future work is multivariable MR, which allows the inclusion of multiple exposures to further decrease the risk of horizontal pleiotropy and provide more extensive testing of causal mechanisms. In addition, triangulating with high-quality observational analyses, or as was done by Davies et al. (2018a) with results from policy reform, would be ideal. There are three important sources of potential bias that are not (sufficiently) accounted for in current MR studies; genetic nurturing (genetic variants that are not transmitted from parents to offspring still affecting offspring phenotype), assortative mating (spouses genetically resembling each other more than by chance because they selected each other based on a genetically influenced trait), and geographic genetic clustering (Brumpton et al., 2020). These phenomena may re-introduce bias from potential confounders, shifting the MR estimate towards the observational association. This can be prevented by performing MR with genetic estimates from within-family GWAS, as these will be corrected for all factors shared within families. Finding large enough family samples will be an important challenge in coming years [the first of such efforts recently became available (Howe et al., 2021). Finally, it is important to acknowledge that almost all MR studies were based on cohorts including participants of European descent. Because of the lack of diversity in the field of genetic research, genetic instruments needed to perform MR for other ethnic groups are rarely available. Increasing diversity in genetic research will be pivotal if we want to reach a comprehensive understanding of the genetic etiology of mental health and substance use, as well as the causal nature of their relationship (Abdellaoui & Verweij, 2021).

## Conclusion

In this systematic review of MR studies, we found strong evidence that higher educational attainment decreases smoking and that there is a bi-directional, increasing relationship between smoking and (symptoms of) mental disorders (depression, bipolar disorder, and schizophrenia). Another robust finding was that higher educational attainment increases alcohol use frequency, whereas it decreases the risk of binge-drinking and alcohol use problems, and that (symptoms of) mental disorders causally lead to more alcohol drinking without evidence for the reverse. Future work should attempt to tackle important limitations that were highlighted in this review. An approach that is particularly noteworthy, and should be used more routinely, is multivariable MR. The etiology of mental health traits is complex and we have only a limited understanding of the biological pathways from SNP to phenotype. It is, therefore, important to test whether key variables act as confounders (inducing a false-positive causal finding) or mediate the causal relationship (i.e. are part of the causal chain from exposure to the outcome). This is especially relevant for MR studies investigating educational attainment as an exposure (McMartin & Conley, 2020). Multivariable MR allows the modeling of complex networks of genetic effects linking different mental health traits. Finally, triangulation of MR results with other research methods is crucial. This includes comparison to other genetically informative methods such as twin studies, latent causal variable analysis (O'Connor & Price, 2018), or genomic structural equation modeling (Grotzinger et al., 2019), carefully conducted longitudinal analyses of cohort data, and/or instrumental variable methods that use environmental factors (e.g. policy changes) instead of genes as an instrument.

Taken together, the current body of MR studies is a valuable addition to the literature on mental health and substance use. It has provided more robust evidence that substance use (most notably smoking) can cause mental health problems, thereby (further) strengthening the incentive to decrease substance use, particularly among populations with poor mental health.

## Supplementary material

For supplementary material accompanying this paper visit https://doi.org/10.1017/S003329172100180X. click here to view supplementary material
