# Supplementary material for: Using Mendelian randomization analysis to better understand the relationship between mental health and substance use: a systematic review
Source: Psychol Med. 2021 May 25;51(10):1593–624. doi: 10.1017/S003329172100180X (PMC8327626; doi:10.1017/S003329172100180X)
Supplement: Supplementary file 1 [file S003329172100180Xsup.zip › S003329172100180Xsup002.docx]

**Supplementary Material to**

**‘Using Mendelian randomization analysis to better understand the relationship between mental health and substance use: A systematic review***’*

*Jorien L Treur, Marcus R Munafò, Emma Logtenberg, Reinout W Wiers, Karin Verweij*

**Content**

Supplemental Methods 1-3

Supplemental Table 1. Quality scoring system 4-6

**Supplemental Methods**

Search terms to identify relevant studies in PsychINFO, Medline, EMBASE, and Web of Science:

**PsycINFO**

*Ovid*

**#1 drugs**

cannabis/ OR hashish/ OR marijuana/ OR marijuana usage/ OR tobacco smoking/ OR smoking cessation/ OR alcohols/ OR alcoholism/ OR alcohol abuse/ OR alcoholic psychosis/ OR alcohol drinking patterns/ OR alcohol intoxication/ OR alcohol withdrawal/ OR drinking behavior/ OR binge drinking/ OR caffeine/ OR (smoking OR tobacco OR cigarette* OR cannabis OR marijuana OR marihuana OR alcohol* OR drink* OR caffeine OR coffee).ti,ab,id

**#2 Mendelian randomization**

mendel* random*.ti,ab,id.

**1 AND 2 53 results (27-02-2020)**

**Medline**

*Ovid MEDLINE(R) and Epub Ahead of Print, In-Process & Other Non-Indexed Citations and Daily 1946 to present*

**#1 drugs**

cannabis/ OR marijuana abuse/ OR marijuana smoking/ OR smoking/ OR smoking reduction/ OR tobacco smoking/ OR "tobacco use disorder"/ OR cigar smoking/ OR cigarette smoking/ OR "tobacco use"/ OR psychoses, alcoholic/ OR alcoholic intoxication/ OR alcoholism/ OR binge drinking/ OR alcohol drinking/ OR drinking behavior/ OR coffee/ OR caffeine/ OR (smoking OR tobacco OR cigarette* OR cannabis OR marijuana OR marihuana OR alcohol* OR drink* OR caffeine OR coffee).ti,ab,kf

**#2 Mendelian randomization**

mendelian randomization analysis/ OR mendel* random*.ti,ab,kf.

**1 AND 2 300 results (27-02-2020)**

**EMBASE**

*Ovid, Embase Classic+Embase 1947 to 2019 January 24*

**#1 drugs**

cannabis/ OR cannabis addiction/ OR cannabis smoking/ OR "cannabis use"/ OR "smoking and smoking related phenomena"/ OR cigarette smoking/ OR smoking cessation/ OR smoking habit/ OR cigar smoking/ OR pipe smoking/ OR smoking reduction/ OR smoking/ OR tobacco smoke/ OR "tobacco use"/ OR tobacco/ OR tobacco dependence/ OR tobacco consumption/ OR alcoholism/ OR alcoholic psychoses/ OR alcoholic intoxication/ OR binge drinking/ OR alcohol/ OR drinking behavior/ OR alcohol consumption/ OR coffee/ OR caffeine/ OR (smoking OR tobacco OR cigarette* OR cannabis OR marijuana OR marihuana OR alcohol* OR drink* OR caffeine OR coffee).ti,ab,kw

**#2 Mendelian randomization**

mendelian randomization analysis/ OR mendel* random*.ti,ab,kw.

**1 AND 2 572 results (27-02-2020)**

**Web of Science**

*Thomson Reuters, Web of Science Core Collection*

**#1 drugs**

TS=("smoking" OR "tobacco" OR "cigarette*" OR "cannabis" OR "marijuana" OR "marihuana" OR "alcohol*" OR "drink*" OR "caffeine" OR "coffee")

**#2 Mendelian randomization**

TS=("mendel* random*")

**1 AND 2 481 results (27-02-2020)**

**Total : 53 + 300 + 572 + 481 = 1,406**

**bioRxiv, medRxiv, and arXiv.org**

Search terms similar to the ones used for the databases above were the basis for our searches in the pre-print servers. Because these pre-print databases lacked a formal systematic search approach, there is no script to share here.

**Supplementary Table 1.** Scoring system to appraise the quality of the included Mendelian randomization (MR) studies

| **Type of method** |  | **-** | **- +** | **+** | **Notes** |
| --- | --- | --- | --- | --- | --- |
| **all** | Phenotype measurement* |  |  |  |  |
|  | **a**. Sample size exposure | <50,000 | 50,000 – 100,000 | >100,000 | *Effective* sample size for case-control studies |
|  | **b**. Sample size outcome | <50,000 | 50,000 – 100,000 | >100,000 | *Effective* sample size for case-control studies |
|  | **c**. Type of measure for exposure | Single or a few (survey) question(s) | Validated, multi-item survey questionnaire | Extensive (cognitive) measure in clinic / clinical diagnosis |  |
|  | **d**. Type of measure for outcome | Single or a few (survey) question(s) | Validated, multi-item survey questionnaire | Extensive (cognitive) measure in clinic / clinical diagnosis |  |
|  |  |  |  |  |  |
|  | Instrument strength** |  |  |  |  |
|  | **a**. *p*-value threshold | ≥5E-08 | <5E-08 | - |  |
|  | **b**. # SNPs included | <3 | 3-10 | >10 | Note that **b** concerns the genetic instruments that include SNPs below the *p-*­value threshold <5E-08 (and *not* additional instruments that include less-significant SNPs ≥5E-08) |
|  | **c**. Biological knowledge of genetic variants | Limited | Reasonable | Good |  |
|  | **d**. F statistic reported | No | Yes | - |  |
|  | **e**. F statistic sufficient | <10 | ≥10 | - |  |
|  | **f**. % variance explained reported | No | Yes | - |  |
|  | **g**. % variance explained sufficient | Low (<1%) | Moderate (1-3%) | High (≥3%) |  |
|  |  |  |  |  |  |
|  | Bidirectional effects tested | No | Yes | - | n.a. when bidirectional causality is not plausible |
|  |  |  |  |  |  |
| **One-sample MR (individual level data)** | Main analysis | Regression analysis (SNP-outcome association) | 2-stage least-squares regression (2SLS) | - |  |
|  |  |  |  |  |  |
|  | Type of genetic instrument | Unweighted allelic score | Weighted allelic score | - |  |
|  |  |  |  |  |  |
|  | Sensitivity analyses |  |  |  |  |
|  | **a**. Method addressing horizontal pleiotropy | No | Yes | - | E.g. testing associations between instrument & potential confounders |
|  | **b**. Leave-one-out analysis or forest plot of  individual SNP-effects | No | Yes | - |  |
|  | **c**. Considerable extra effort with additional  sensitivity method(s) | No | - | Yes | E.g. using negative / positive controls or directly comparing findings to another, *non*-MR research method (triangulation) |
|  |  |  |  |  |  |
| **Two-sample MR (summary level data)** | Appropriate temporality | No | Yes | - | If trait 1 is measured in childhood GWAS and trait 2 in adulthood GWAS, then causality from trait 2 🡪 trait 1 can’t logically be tested |
|  | Mention of harmonization of genetic variants across the datasets | No | Yes |  | Should be scored as ‘-+’ if harmonization is explicitly mentioned by the authors or when MR Base was used |
|  |  |  |  |  |  |
|  | Estimates SNP-exposure and SNP-outcome are from the same ethnic group | No | Yes | - |  |
|  |  |  |  |  |  |
|  | Sample overlap |  |  |  |  |
|  | **a**. Is % overlap reported | No | Yes | - |  |
|  | **b**. Is there sample overlap | Yes | No | - | Should be considered especially problematic if sample overlap is high |
|  |  |  |  |  |  |
|  | Sensitivity analyses |  |  |  |  |
|  | **a**. Addressing horizontal pleiotropy | No | Yes | - | E.g. MR-Egger which explicitly estimates horizontal pleiotropy or an approach that excludes or down-weights outlier SNPs |
|  | **b**. Leave-one-out analysis or forest plot of  individual SNP-effects | No | Yes | - |  |
|  | **c**. Considerable extra effort with additional  sensitivity method(s) | No | - | Yes | E.g. using negative / positive controls, directly comparing findings to another, non-MR research method (triangulation), or using many different MR sensitivity methods with contrasting underlying assumptions |

SNP = Single Nucleotide Polymorphism, GWAS = Genome-Wide Association Study. Note: where absolute thresholds are used to judge the quality of a particular aspect of the study (e.g. sample size), it should be noted that these are somewhat arbitrary and were merely used to provide an indication of quality. The formula used to calculate the effective sample size for case-control studies = *4 / (1 / ncases + 1 / ncontrols).* Regarding sample overlap, note that we only indicate whether or not there is (some) overlap between samples, and not the amount, given that there is no reliable threshold from which to infer the potential effects of overlap on causal estimates. In addition, the percentage of overlap should be taken with respect to the larger dataset (e.g., if the smaller dataset has 1,000 individuals and all of these individuals are also in the larger dataset of 10,000 individuals, sample overlap is 10%) as indicated by Burgess et al., 2016, *Genetic Epidemiology*.*With regards to ‘Phenotype measurement’, a very well measured phenotype in a moderate sample size may be just as powerful as a more superficially measured phenotype in a very large sample. However, in case of very small sample sizes (e.g. n = 180 such as in the study by Irons et al., 2007) even an extremely thoroughly measured phenotype will not lead to a high total score. **With regards to ‘Instrument strength’, when a study uses a single genetic variant that explains a relatively large amount of the variance and for which there is good biological knowledge, the fact that only 1 SNP was used is not necessarily problematic. For example, this is the case for SNP rs1051730 in the nicotinic acetylcholine receptor *CHRNA5/A3/B4* gene cluster – each additional risk allele increases smoking heaviness with 1 additional cigarette smoked per day (Katikireddi SV et al., 2018).
